# High temporal resolution Nanopore sequencing dataset of SARS-CoV-2 and host cell RNAs

**DOI:** 10.1093/gigascience/giac094

**Published:** 2022-10-17

**Authors:** Dóra Tombácz, Ákos Dörmő, Gábor Gulyás, Zsolt Csabai, István Prazsák, Balázs Kakuk, Ákos Harangozó, István Jankovics, Béla Dénes, Zsolt Boldogkői

**Affiliations:** Department of Medical Biology, Albert Szent-Györgyi Medical School, University of Szeged, Szeged 6720, Hungary; Department of Medical Biology, Albert Szent-Györgyi Medical School, University of Szeged, Szeged 6720, Hungary; Department of Medical Biology, Albert Szent-Györgyi Medical School, University of Szeged, Szeged 6720, Hungary; Department of Medical Biology, Albert Szent-Györgyi Medical School, University of Szeged, Szeged 6720, Hungary; Department of Medical Biology, Albert Szent-Györgyi Medical School, University of Szeged, Szeged 6720, Hungary; Department of Medical Biology, Albert Szent-Györgyi Medical School, University of Szeged, Szeged 6720, Hungary; Department of Medical Biology, Albert Szent-Györgyi Medical School, University of Szeged, Szeged 6720, Hungary; Complex Medical Center, Budapest 1012, Hungary; Veterinary Diagnostic Directorate, National Food Chain Safety Office, Budapest 1143, Hungary; Department of Medical Biology, Albert Szent-Györgyi Medical School, University of Szeged, Szeged 6720, Hungary

**Keywords:** SARS-CoV-2, coronavirus, long-read sequencing, full-length transcriptome, Oxford Nanopore Technologies, MinION system, direct RNA sequencing, direct cDNA sequencing

## Abstract

**Background:**

Recent studies have disclosed the genome, transcriptome, and epigenetic compositions of severe acute respiratory syndrome coronavirus 2 (SARS-CoV-2) and the effect of viral infection on gene expression of the host cells. It has been demonstrated that, besides the major canonical transcripts, the viral genome also codes for noncanonical RNA molecules. While the structural characterizations have revealed a detailed transcriptomic architecture of the virus, the kinetic studies provided poor and often misleading results on the dynamics of both the viral and host transcripts due to the low temporal resolution of the infection event and the low virus/cell ratio (multiplicity of infection [MOI] = 0.1) applied for the infection. It has never been tested whether the alteration in the host gene expressions is caused by aging of the cells or by the viral infection.

**Findings:**

In this study, we used Oxford Nanopore's direct cDNA and direct RNA sequencing methods for the generation of a high-coverage, high temporal resolution transcriptomic dataset of SARS-CoV-2 and of the primate host cells, using a high infection titer (MOI = 5). Sixteen sampling time points ranging from 1 to 96 hours with a varying time resolution and 3 biological replicates were used in the experiment. In addition, for each infected sample, corresponding noninfected samples were employed. The raw reads were mapped to the viral and to the host reference genomes, resulting in 49,661,499 mapped reads (54,62 Gbs). The genome of the viral isolate was also sequenced and phylogenetically classified.

**Conclusions:**

This dataset can serve as a valuable resource for profiling the SARS-CoV-2 transcriptome dynamics, the virus–host interactions, and the RNA base modifications. Comparison of expression profiles of the host gene in the virally infected and in noninfected cells at different time points allows making a distinction between the effect of the aging of cells in culture and the viral infection. These data can provide useful information for potential novel gene annotations and can also be used for studying the currently available bioinformatics pipelines.

## Data Description

### Background

Severe acute respiratory syndrome coronavirus 2 (SARS-CoV-2) is a positive-sense single RNA-stranded betacoronavirus and the etiological agent of the current coronavirus disease 2019 (COVID-19) pandemic [1]. The replication and the transcription of the RNA genome are interrelated because the same enzyme, an RNA-dependent RNA polymerase (RdRP), carries out both processes [2]. First, negative-sense RNA intermediates are generated to serve as templates for the synthesis of both the genomic RNA (gRNA) and the nested set of subgenomic RNAs (sgRNAs) [3]. The gRNA and the sgRNAs have common 5′- and 3′-termini since the RdRP synthesizes the positive sense RNAs from this end of the genome. Template switching occurs during the synthesis of the negative strand of sgRNAs, which is mediated by the transcription-regulating sequences (TRSs) in the genome body (TRS-B) and in the 5′-leader sequence (TRS-L), resulting in the fusion of leader–body sequences [4, 5]. Recent studies have disclosed the transcriptomic architecture of SARS-CoV-2 and the effect of viral infection on the host gene expression [6]. It has been shown that, besides canonical TRS-dependent RNA molecules, the viral genome also codes for noncanonical, TRS-dependent, and TRS-independent transcripts, although in a relatively low abundance (altogether <10%). Additionally, investigations of the effect of the viral infection on the transcriptome of various cell types have identified several genes and gene networks [7].

Nonetheless, the kinetic studies of gene expressions used only a few time points for monitoring the infection [8, 9], which do not provide a comprehensive picture on the temporal dynamics of the viral transcriptome. Furthermore, typically, a low (0.1) multiplicity of infection (MOI) was applied in the experiments, which may lead to misleading conclusions on the kinetic properties of SARS-CoV-2 transcripts, because after the completion of the replication cycle, the virus can gradually initiate new infection cycles within the noninfected cells [10]. Low MOI infection also makes it difficult to assess the host cell response, especially in the case of the downregulated genes. Infections are typically carried out using fresh, rapidly growing cultured cells, but only the fresh cells (at time point 0) are used as mock-infected cells. Nonetheless, gene expression profiles may undergo alterations in noninfected cells during the propagation; therefore, we cannot decide whether the transcriptional changes in infected cells are due to the effect of the virus or to the time factor of culturing (aging of cells). This phenomenon has practically never been tested in the experiments. An additional problem is the use of short-read sequencing for profiling of the host cell reaction to the viral infection [7] because this approach has severe limitations for the detection of transcript isoforms, such as splice and length variants, and multigenic transcripts, among others [11–13].

Long-read sequencing (LRS) opened new avenues for the comprehensive analysis of the transcriptomes, for which the major reason is that these techniques are able to detect full-length RNA molecules and thereby to distinguish between transcript isoforms and transcriptional overlaps. LRS-based studies have revealed a hidden transcriptional complexity in viruses [14–17], but this approach has also been used for the analysis of the kinetic properties of viral transcriptomes [18], the analysis of RNA modifications [16, 19], and the virus–host interaction [20, 21].

In this study, we applied Nanopore sequencing based on direct RNA (dRNA) and direct cDNA (dcDNA) approaches for the generation of transcriptomic datasets from SARS-CoV-2 and primate host Vero cells. A mixed time-point sample (single library from a mixture containing equal amount of total RNAs from each of the 16 time points) was used for dRNA sequencing, while we used 16 time-point samples within an interval of 1 to 96 hours from both infected and noninfected host cells using MOI = 5 for the infection.

Decoding the transcriptional landscape of SARS-CoV-2 virus is a fundamental step in studying its biology, genetic regulation, and molecular pathogenesis. Therefore, in this data descriptor, our aim was to provide a robust, precise, reliable dataset based on LRS approaches for understanding the gene expression and genetic regulation of the causative agent of the current pandemic, as well as its effect on differential host gene expression, and to provide a rich resource for future functional studies.

### Methods

**Figure 1:**
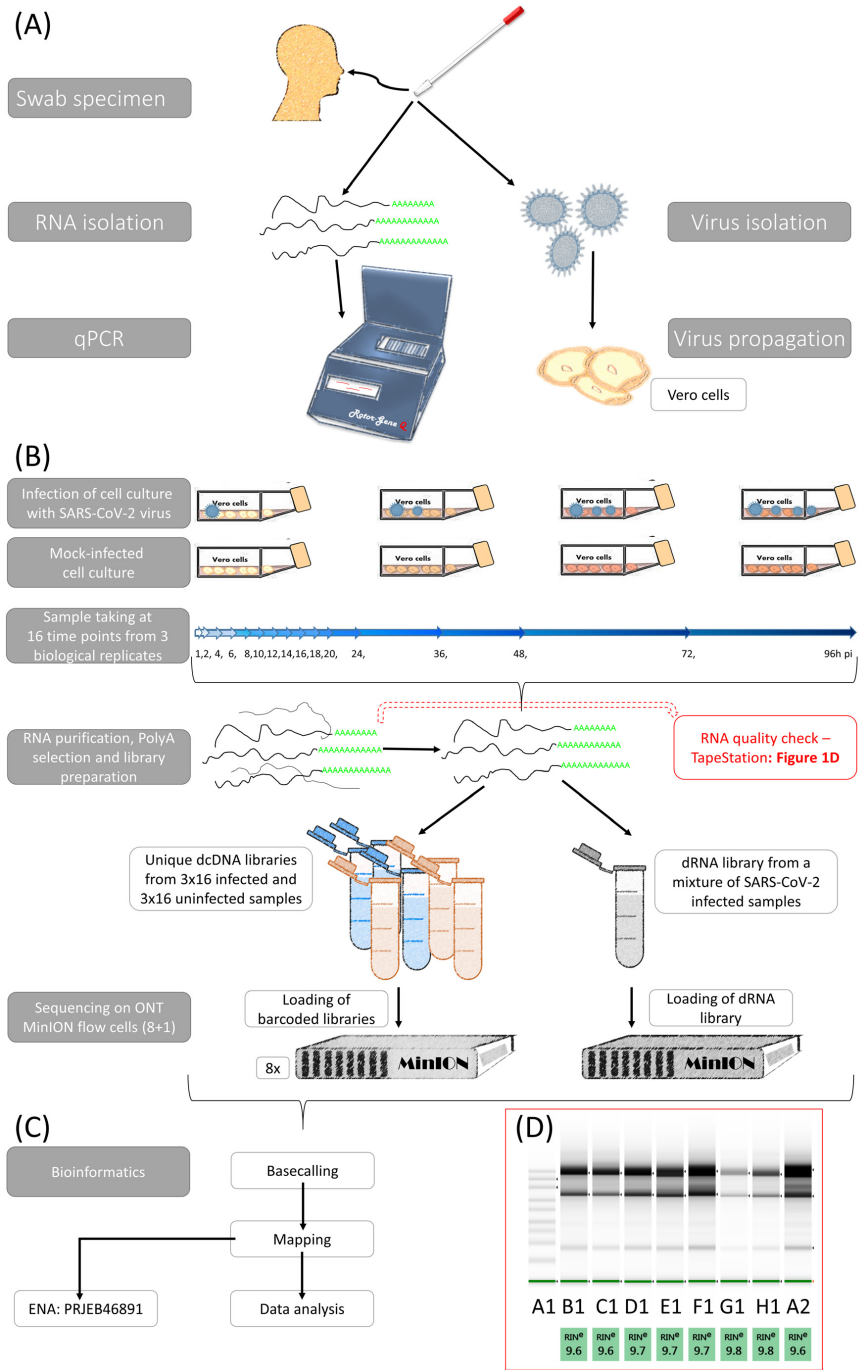
Schematic representation of the workflow applied in this project. (A) Isolation and detection of a Hungarian isolate of the SARS-CoV-2 virus. The sample was collected from a human nasopharyngeal swab. The SARS-CoV-2 infection was validated by reverse transcription PCR using the RNA extracted from the sample. The virus was isolated from the sample and was maintained on Vero cells. (B) Experimental workflow of the study. Vero cells were infected with SARS-CoV-2 and the cells were incubated at 37°C for 1, 2, 4, 6, 8, 10, 12, 14, 16, 18, 20, 24, 36, 48, 72, and 96 hours post infection. Uninfected control cells were also propagated. Each time-point experiment was carried out in 3 biological replicates. RNAs were purified from the samples, which was followed by the preparation of libraries and then sequencing using direct cDNA and direct RNA methods. Altogether, 9 MinION flow cells (ONT) were used for this study. (C) Bioinformatics workflow. The ONT's Guppy basecaller was used to identify the base sequence of the obtained reads, and then they were aligned to the viral and host reference genomes by using the minimap2 mapper. Statistical data were generated with seqtools [25] and a custom R-workflow [33]. (D) Quality of RNA samples was detected with a TapeStation 2200 system with RNA ScreenTape. TapeStation gel image shows that intact, high-quality RNAs were isolated from the samples and used for sequencing. The image shows the following samples: A1: marker; B1: 8-hour postinfection (pi) sample C; 12-hour pi sample A; 16-hour pi sample A; 18-hour pi sample B, 20-hour pi sample C; 36-hour pi sample A; 48-hour pi sample A; 96-hour pi sample B.

Fig. 1 shows the detailed workflow of the study.

#### Cells

The Vero E6 (African green monkey kidney) cell line was obtained from the American Type Culture Collection (ATCC). The cells were plated at a density of 2 × 10^6^ cells per 75-cm^2^ tissue culture flasks (CELLSTAR®; Greiner Bio-One GmbH, Frickenhausen, Germany) in Minimum Essential Medium Eagle culture medium (MEM) with 10% fetal bovine serum (FBS) and 2 mM L-glutamine and antibiotic-antimycotic solution (all obtained from Sigma-Aldrich, Burlington, MA). Vero cells were incubated at 37°C in a humidified 5% CO_2_ atmosphere until confluency (∼8 × 10^6^ cells) was reached. The monolayer was washed once with the serum-free MEM immediately before infection.

#### Collection, detection, and isolation of the virus

The SARS-CoV-2 virus was isolated from the human nasopharyngeal swab of the reverse transcription PCR (RT-PCR)–positive (Ct 22) 77-year-old male patient during the official COVID-19 surveillance program at the Veterinary Diagnostic Directorate of the National Food Chain Safety Office (Budapest, Hungary) with the cooperation of the Complex Medical Center (Budapest) in November 2020 during the second wave of the COVID-19 pandemic in Hungary. The patient developed respiratory illness, with fever, cough, and fatigue that quickly progress edto pneumonia. The patient was hospitalized, where, unfortunately, he died in a few days. In his story, he did not declare any travel abroad in the last 14 days. At the same time, he traveled relatively frequently within Hungary and had been in close contact with people who had COVID-19.

Detection of SARS-CoV-2 in pharyngeal wash samples was performed using RT-PCR amplification of SARS-CoV-2 N-gene fragments. In total, 200 μL of the pharyngeal washes was first processed for RNA extraction in the Thermo Scientific™ KingFisher™ Flex Purification System (Thermo Fisher Scientific, Waltham, MA, USA), using the IndiMag® Pathogen Kit (QIAGEN® GmbH, Hilden, Germany). Subsequently, the detection of N-gene of SARS-CoV-2 was performed by using the 2019-nCoV-2 RUO kit (Integrated DNA Technologies, Inc., Coralville, IA, USA) and One-Step RT-PCR Kit (QIAGEN® GmbH) on a Rotor-Gene Q real-time PCR cycler (QIAGEN® GmbH). The amplification protocol consisted of a reverse transcription step at 50°C for 30 minutes, a denaturation step at 95°C for 15 minutes, and subsequent 45 cycles at 95°C/56°C/72°C for 30/30/60 seconds, respectively. A positive result was defined as amplification of N-gene in a sample with each cycle threshold value (ct) less than 37.

For the virus isolation, 1 mL viral transport media from the swab was mixed with 3 mL serum-free MEM culture medium supplemented with 2 mM L-glutamine and antibiotic-antimycotic solution and filtered using a Ministar® 0.22-μm filter (Sartorius AG, Göttingen, Germany). The filtrate was placed onto cells in a 25-cm^2^ tissue culture flask (Corning®; Corning, Inc., New York, NY, USA) of Vero E6 cells, then incubated at 37°C in a humidified 5% CO_2_ chamber for 1 hour. After incubation, 2.5 mL serum-free MEM culture medium with 2 mM L-glutamine and antibiotic-antimycotic solution was added to the tissue culture flask. The inoculated culture was grown in a humidified 37°C incubator with 5% CO_2_. Cells were observed daily for cytopathic effect (CPE). On day 4, an 80% CPE was evident, and the cells with supernatant were harvested. This provided the first-passage virus. The virus was passaged twice at low MOI in Vero E6 cells to obtain a working stock used in the experiments. The viral titer was determined by plaque assay on Vero E6 cells. Virus stock was stored at −80°C until use.

#### Propagation of the virus

The virus was passaged twice in Vero cells to obtain a working stock used in all experiments. Viral titer was determined by plaque assay on Vero cells. The virus was diluted into a serum-free MEM. Cells were infected with 5 mL SARS-CoV-2 virus with 5 plaque-forming units (pfu)/cell (MOI = 5), then incubated at 4°C for 1 hour. Noninfected control cultures (mock) were prepared using pure nonsupplemented MEM as inoculums. Next, the virus inoculum was removed from the flasks. The monolayer was washed once with the serum-free MEM. Then, 10 mL MEM culture medium supplemented with 3% FBS, 2 mM L-glutamine, and antibiotic-antimycotic solution was added to the tissue culture flasks. The cells were incubated at 37°C for 1, 2, 4, 6, 8, 10, 12, 14, 16, 18, 20, 24, 36, 48, 72, and 96 hours in a humidified 5% CO_2_ atmosphere. Each time, the experiment was done in triplicate with a mock-infected control. Mock-infected cells were harvested at the same time points as the infected cells. Following incubation, the medium was removed, and the monolayer was washed once with phosphate-buffered saline (PBS). The tissue culture plates were stored at −80°C until use. Next, the infected cells were treated by lysis buffer, then creped and placed into an Eppendorf Tubes^®^ (Thermo Fisher Scientific, Inc.).

#### RNA purification

Total RNA was extracted from the mock-infected and SARS-CoV-2–infected cells at various stages of infection from 1 to 96 hours using Macherey-Nagel's NucleoSpin RNA Kit, Düren, Germany according to the manufacturer's protocol. In brief, cells were collected by low-speed centrifugation, and then 350 μL lysis buffer (RA1 from the kit) and 3.5 μL β-mercapthoethanol (Sigma-Aldrich, St. Louis, MO, USA) were added followed by vortexing the samples. Mixtures were loaded onto a NucleoSpin Filter and centrifuged for 1 minute at 11,000 × *g*. The filters were discarded and 350 μL 70% EtOH was added to the lysate. This was loaded to the NucleoSpin RNA Column and centrifuged at 11,000 × *g* for 30 seconds. Membrane was desalted with the addition of 350 μL Membrane Desalting Buffer (from the NucleoSpin Kit), then dried with a short centrifugation (11,000 × *g*). Residual DNA was enzymatically removed (with usage of the 95-μL mixture of rDNase/rDNase reaction buffer [1:9 ratio, NucleoSpin Kit] and incubation at room temperature [RT] for 15 minutes). The rDNase was inactivated with the first washing step by adding 200 μL RAW2 Buffer (NucleoSpin Kit) directly onto the NucleoSpin Filter. After a quick centrifugation (30 minutes, 11,000 × *g*), the filter was placed in a new tube. Then, 600 μL RAW3 Buffer (NucleoSpin Kit) was added and spun down as before. This washing step was repeated using 250 μL RAW3. Finally, the total RNA bound to the filter was eluted in 60 μL nuclease-free water (NucleoSpin Kit). Samples were quantified by Qubit 4.0 using the Qubit RNA BR Assay Kit (Invitrogen, Carlsbad, CA, USA; Supplementary Table S3A) and then stored at −80°C until use.

#### Poly(A) selection

The Poly(A) RNA Selection Kit V1.5 (Lexogen, Wien, Austria) was used to isolate polyadenylated RNAs from the total RNA samples. The protocol applies oligo(dT) beads, which capture RNAs with poly(A) stretches (most mRNAs), but RNAs without polyadenylated 3′ ends (e.g., 28S and 18S rRNAs and tRNAs) do not hybridize to the beads, and therefore, they will be removed during the washing steps. The detailed protocol is as follows: the magnetic beads (part of the Lexogen kit) were resuspended and 4 μL for each RNA sample was measured. Beads were placed in a magnet; they were collected and the supernatant was discarded. Samples were resuspended in 75 μL Bead Wash Buffer (Lexogen kit) and then were placed on the magnet. Supernatant was discarded, and this washing step was repeated once. Beads were resuspended in 20 μL RNA Hybridization Buffer (part of the Lexogen kit). Then, 10 ng from the total RNA samples was diluted to 20 μL UltraPure™ DNase/RNase-Free Distilled Water (Invitrogen) and then denatured at 60°C for 1 minute, followed by holding them at 25°C. Next, 20 μL denatured RNA was mixed with 20 μL (previously washed and resuspended) beads. The mixtures were incubated at 25°C in a shaker incubator with 1,250 rpm agitation. After a 20-minute incubation, sample-containing tubes were placed in a magnetic rack. Supernatant was discarded, and then the tubes were removed from the magnet. Samples were resuspended in 100 μL Bead Wash Buffer (Lexogen kit), and then they were incubated for 5 minutes at 25°C with 1,250 rpm agitation. Supernatant was discarded and the washing step was repeated. After the complete removal of the supernatant, beads were resuspended in 12 μL UltraPure™ DNase/RNase-Free Distilled Water. Samples were incubated at 70°C for 1 minute, and then the tubes were placed on a magnetic rack. Supernatant containing the poly(A)+ RNA fraction was placed to new DNA LoBind (Eppendorf) tubes, the RNA concentration was measured using the Qubit RNA HS Assay Kit (Invitrogen, Supplementary Table S3B), and then samples were stored at −80°C.

#### ONT—direct cDNA sequencing

For the analysis of the dynamic properties of SARS-CoV-2 RNAs and the effect of viral infection on the host cell transcriptome profile, RNA samples from different time points (1, 2, 4, 6, 8, 10, 12, 14, 16, 18, 20, 24, 36, 48, 72, and 96 hours postinfection [pi]; Supplementary Table S3C) were used individually for the generation of direct cDNA libraries for Nanopore sequencing. The nonamplified cDNA libraries were prepared from 16 time points from the mock and coronavirus-infected samples in 3 biological replicates using the Direct cDNA Sequencing Kit (SQK-DCS109; ONT, Oxford, England) and the appropriate ONT protocol. In short, first-strand cDNAs were generated from the polyA(+) RNAs using the Maxima H Minus Reverse Transcriptase (Thermo Fisher Scientific) with SSP and VN primers (supplied in the kit). The RNase Cocktail Enzyme Mix (Thermo Fisher Scientific) was used to eliminate the potential RNA contamination. Synthesis of the second cDNA strands was carried out with LongAmp Taq Master Mix (New England Biolabs, Ipswich, MA). The double-stranded cDNAs were repaired (NEBNext End repair/dA-tailing Module; New England Biolabs) and adapter ligated (NEB Blunt/TA Ligase Master Mix; New England Biolabs). Individual barcode sequences were added to each sample for multiplex sequencing, for which the Native Barcoding (12) Kit (ONT) was used as recommended by the manufacturer. The cDNAs and the libraries were washed using AMPure XP beads (Agencourt; Beckman Coulter, Brea, CA) after every enzymatic reaction step. The barcode-labeled samples were loaded onto MinION R9.4 SpotON Flow Cells (ONT; Table 2).

#### ONT—direct RNA sequencing

ONT's Direct RNA sequencing (SQK-RNA002; Version: DRS_9080_v2_revO_14Aug2019, Last update: 10/06/2021) was used to sequence the native RNA strands from a mixture of polyA(+) RNA fractions (Supplementary Table S3D). In total, 500 ng RNA in 9 μL nuclease-free water was mixed with 3 μL NEBNext Quick Ligation Reaction Buffer (New England Biolabs), 0.5 μL RNA CS (ONT kit), 1 μL RT Adapter (110 nM; ONT kit), and 1.5 μL T4 DNA Ligase (2 M U/mL; New England Biolabs). The ligation reaction was carried out for 10 minutes at RT. The synthesis of the first-strand cDNA was conducted using SuperScript III Reverse Transcriptase (Life Technologies), as described in the Direct RNA sequencing (DRS) protocol (ONT). In short, a 50-minute incubation at 50°C was followed by the inactivation of the enzyme at 70°C for 10 minutes. Sequencing adapters from the DRS kit were ligated to the cDNA with the T4 DNA ligase enzyme and NEBNext Quick Ligation Reaction Buffer (New England Biolabs). Ligation was carried out at RT for 10 minutes. The sample was washed using AMPure XP beads (Agencourt; Beckman Coulter) after every enzymatic reaction. Libraries were sequenced on an R9.4 SpotON Flow Cell.

#### Technical validation

***RNA***. The Qubit RNA BR Assay Kit (Invitrogen) was used to check the amount of total RNA. Qubit RNA HS Assay Kit (Invitrogen) was used to measure the quantity of the poly(A)+ RNA fractions. The final concentrations of the RNA samples were determined by Qubit^®^ 4.

***cDNA*.** The concentrations of the cDNA samples and sequencing-ready libraries were measured using the Qubit dsDNA HS Assay Kit (Invitrogen). The quality of RNA was assessed using the Agilent (Santa Clara, CA) 2200 TapeStation System. RIN scores ≥9.6 were used for sequencing (Fig. 1D).

The cDNAs and the sequencing-ready cDNA libraries were washed using AMPure XP beads (Agencourt; Beckman Coulter) after every enzymatic reaction. The samples for dRNA sequencing were treated with RNAClean XP beads.

Three biological replicates were used for each of the 16 time points. To monitor the effect of SARS-CoV-2 infection on the gene expression of the host cells, mock-infected cells were harvested at the same time points, as the virally infected cells.

### Data analysis

The MinION raw data were basecalled using ONT Guppy basecalling software version 5.0.11 using –qscore_filtering: reads with a Q-score of 8 or greater were termed “passed” and those below were termed “failed.” The VirStrain [22] tool was used on the “passed” reads to identify the closest SARS-CoV-2 strains to our isolate (Supplementary Fig. S1 and Supplementary Information S1). The resulting most likely genome (NCBI nucleotide accession: MT560672.1) was used as reference for the mapping of the reads. The infected samples reads were mapped to the host (*Chlorocebus_sabeus* 1.1) genome (GenBank assembly accession: GCA_000409795.2) as well, while the mock (uninfected) samples were mapped to the host genome only. The mappings were carried out with the *minimap2* aligner [23], using the following parameters: *minimap2 -ax splice -Y -C5*. The *view* command from the SamTools package [24] was used on the resulting “sam” files to generate binary alignment (“.bam”) files, which were subsequently sorted and indexed, using the *sort* and *index* commands, respectively; finally, the *view* command was used again to separate the data into viral-mapped, host-mapped, and unmapped “.bam” files. Our in-house developed Python script “readstats.py” was used to generate the descriptive statistics of reads and the alignments [25]. The output of *the readstat* script, containing the mapping statistics, was imported into R. Subsequently, the median, 25th percentile, and 75th percentile values of the mapped read lengths were calculated and visualized using ggplot2 [26] for both the viral and host reads (Fig. 5). In the case of the infected samples, the ratios of the reads mapped to the viral and host genome were also visualized using ggplot2 (Supplementary Fig. S3).

**Figure 2:**
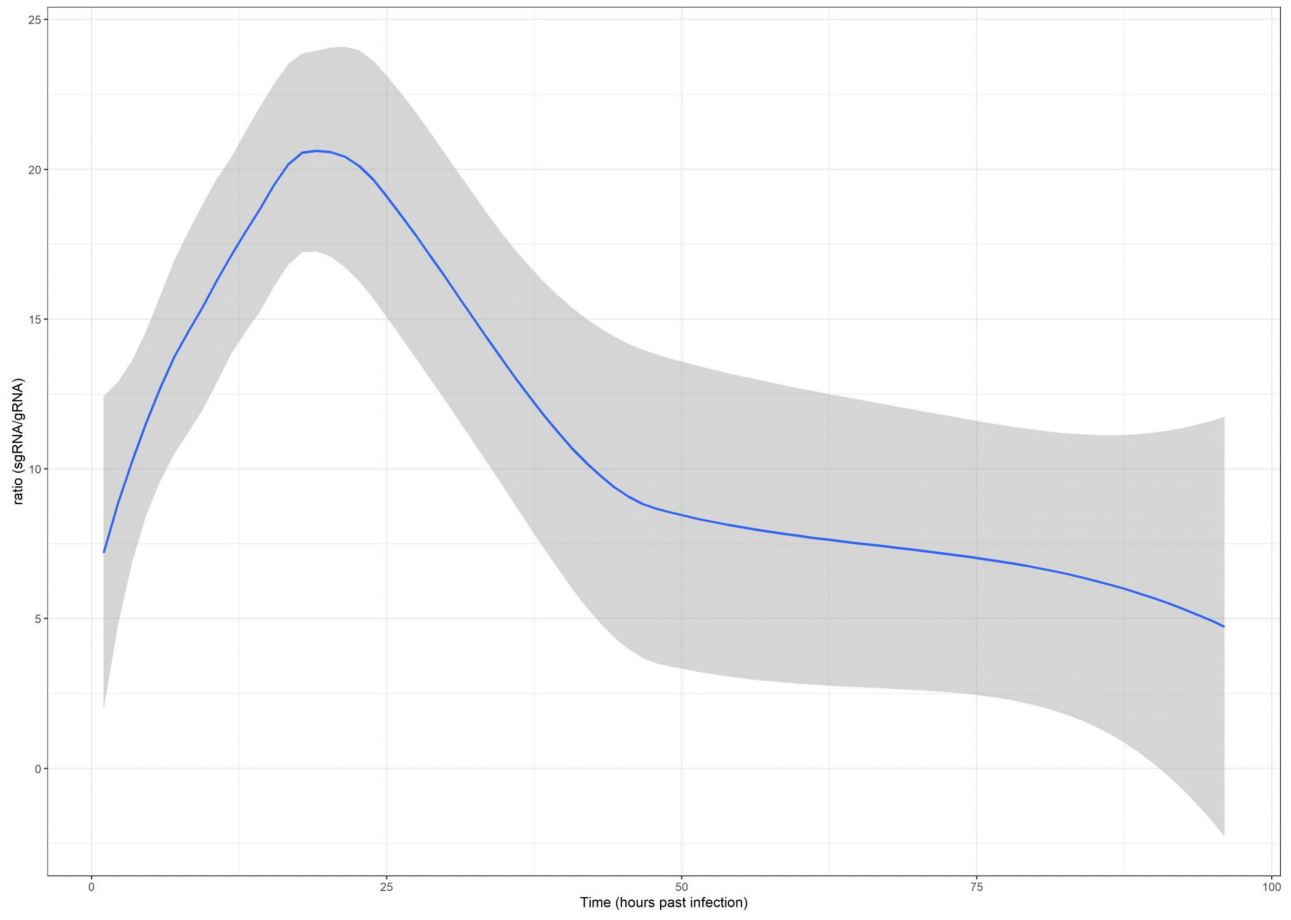
Ratio of the sgRNAs to the gRNAs across the viral infection cycle in the dcDNA samples. The fitted loess function with 95% confidence intervals is shown in blue and gray, respectively.

To distinguish RNAs originating from the viral gRNA from the sgRNA transcripts, we further processed the reads by remapping the reads initially mapped to the original Wuhan genome isolate (NC_145512.2) with *minimap2 -ax splice -Y -C5 –MD -un -g 30 000 -G 30 000 -O2,24 -E1,0 -C0 -z 400 200 –no-end-flt -F 40 000 –secondary = no –splice-flank = no –max-chain-skip = 40 –for-only*. The alignments were subsequently imported into R and processed via an in-house developed script, utilizing packages of the tidyverse [27], RSamtools [28], GenomicAlignments [29], tidygenomics [30], and dplyr [31]. Subgenomic RNAs were defined as RNAs that overlap with either subgenomic ORF and have a template switch, connecting this mapped region with the 5′-leader part of the genome (in the 55–85 position of the reference genome). Genomic RNAs were defined as those RNAs that overlap with ORF1ab (with at least 10 nt) and are not in the subgenomic category. All other reads were categorized as “unclassified” RNAs. The ratio of the subgenomic/genomic categories in each sample was visualized in a scatterplot with a fitted loess function (Fig. 2).

From the imported alignments, genome coverage was calculated and subsequently visualized in a log_10_ scale (Fig. 4) using ggplot2 [26], and the ORF annotations were generated with *gggenes* [32].

The mapped parts of the RNAs were summed to calculate transcript lengths. From these data, violin plots were generated for the genomic, subgenomic, and unclassified RNAs as well (Supplementary Fig. S1).

The scripts that were used to analyze the alignments and to classify them as genomic or subgenomic origin is available as a complete workflow, that is, from downloading the reads to generate the figures, at a GitHub repository [33]. The R-scripts can be used with other *bam* files, reference genomes, and/or parameters, as well to import, filter, and analyze alignments or to dereplicate them into transcripts.

**Figure 3:**
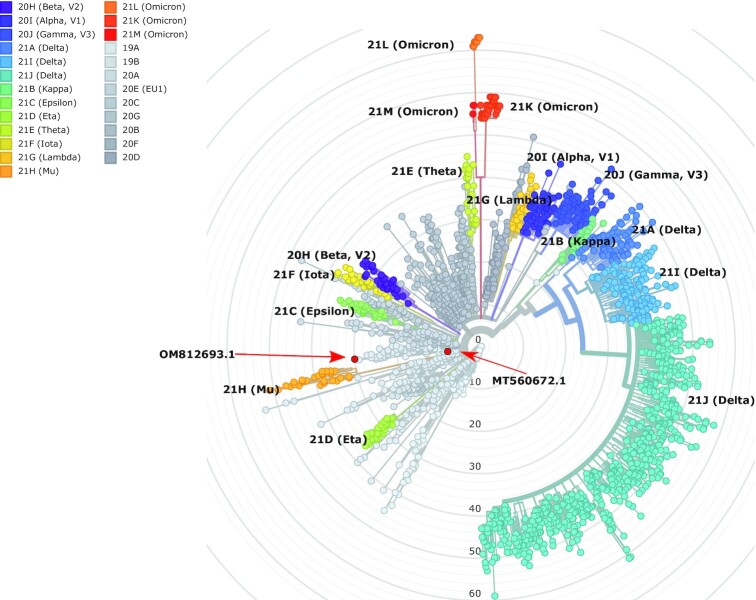
Phylogenetic tree displays the sequenced SARS-CoV-2 strains, according to the designated clades of the virus. Our isolate is colored red, and a red arrow shows the position of our own isolate documented in the current study (OM812693.1). The position of the genome that was used as reference for aligning the reads (MT560672.1) is also indicated by a red arrow. The tree was generated by the Nextstrain pipeline. All variants are colored by their assigned clade, according to the nomenclature.

The SARS-CoV-2 genome was assembled with the shasta program (v.0.9.0) [34] using all viral reads longer than 20,000 bps (*shasta –Reads.minReadLength 20 000 –config Nanopore-Oct2021*; otherwise default parameters). The obtained draft assembly (SARS-CoV-2_Hun-1_GenomeDraft_v1) was analyzed for mutations and characterized phylogenetically with the Nextstrain [35] program, along with the genome from the VirStrain result (Fig. 3; Supplementary Fig. S2; Supplementary Information S1, S2, and S3; and Supplementary Table S4). The draft assembly was submitted to NCBI (sequence accession: OM812693.1).

**Figure 4:**
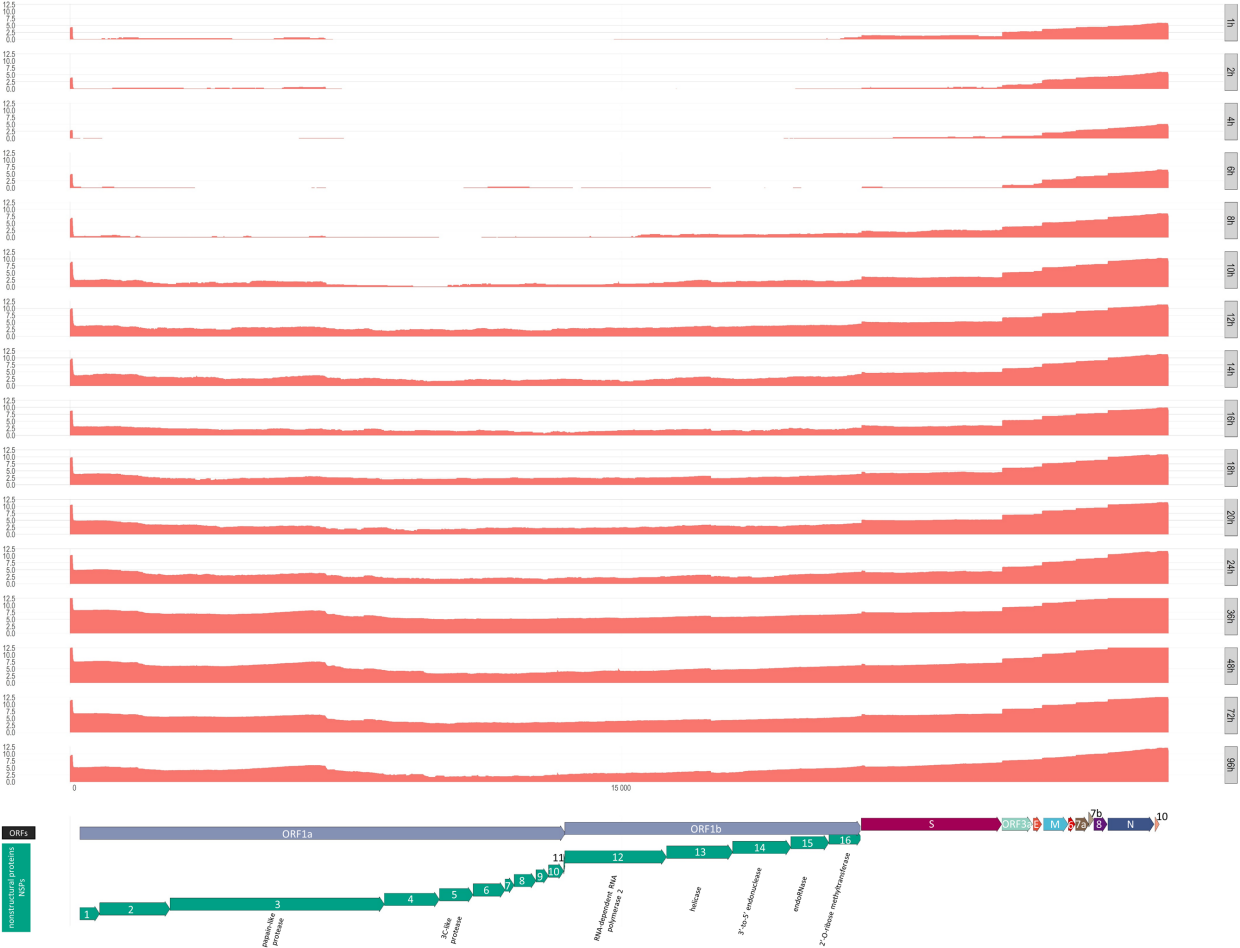
Whole-genome coverage plot using high-quality (Q-score ≥8) reads from dcDNA samples that aligned to the SARS-CoV-2 genome used as a reference for this study. The coverages of the replicates from each hours post infection (hpi) group were summed, and the y-axes show the log_2_ of these values. Annotated protein-coding genes are shown at the bottom track. Direction of arrows depicts the coding strand.

**Figure 5:**
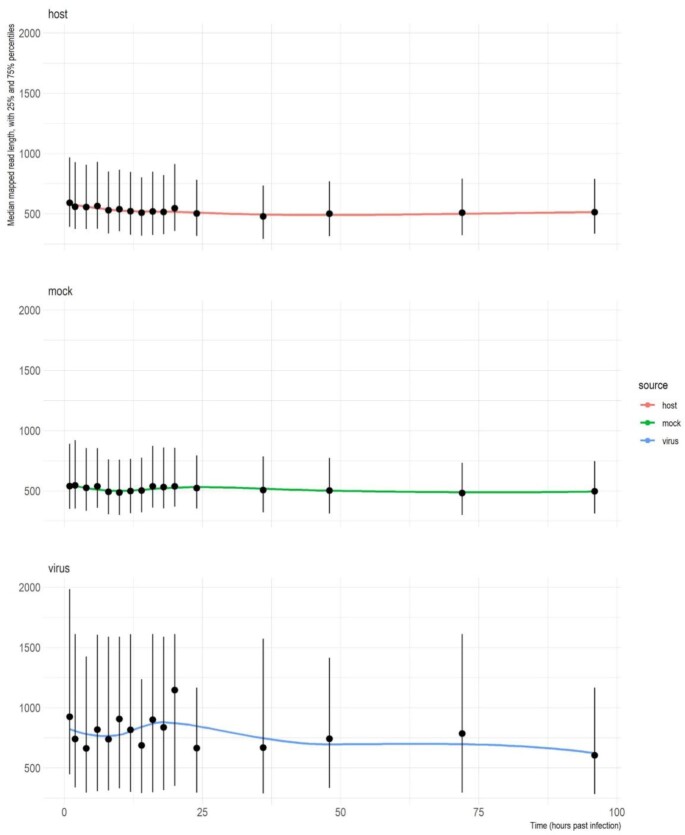
Scatterplot of mean read lengths of the sequencing data derived from infected and uninfected samples, with 25th and 75th percentiles and a fitted loess function. (A) Length of reads aligned to the viral (B) and to the host genome. (C) Read-length distribution of mock-infected samples mapped to the host genome.

Altogether, we generated almost 64 million long reads from which more than 1.8 million reads mapped to the SARS-CoV-2 and almost 48 million to the host reference genome (Table 1). Time course changes in the virus-to-host ratio is depicted in Supplementary Fig. S3. The obtained read count resulted in a very high coverage across the viral genome (Fig. 4). Detailed data on the read counts; quality of reads, including read lengths (Fig. 5); insertions; deletions; and mismatches are summarized in Supplementary Tables S1A, B and S2A, B.

**Table 1:** Summary data of the obtained read counts from dcDNA and dRNA sequencings. Low-quality (failed) reads (Q-score <8) were filtered out from the passed reads (Q-score ≥8) by the MinKNOWs (Guppy, ONT) software.

	Quality	Total (infected)	Virus	Host (infected)	Unmapped (infected)	Total (uninfected)	Host (uninfected)	Unmapped (uninfected)
dcDNA	**all**	**32,017,113**	**1,527,249**	**23,703,827**	**6,786,037**	**29,294,533**	**22,149,844**	**7,144,689**
dcDNA	≥8	23,607,200	1,280,395	21,246,856	1,079,949	20,360,096	19,008,016	1,352,080
dcDNA	<8	8,409,913	246,854	2,456,971	5,706,088	8,934,437	3,141,828	5,792,609
dRNA	**all**	**2,606,502**	**281,418**	**1,999,161**	**325,923**	—	—	—
dRNA	≥8	1,950,595	236,518	1,658,588	55,489	—	—	—
dRNA	<8	655,907	44,900	340,573	270,434	—	—	—

The bold row represent the sum of the two rows below that.

**Table 2:** List of the sequences of barcodes used for multiplex sequencing. This table also contains the information about the barcoded samples loaded on the same flow cell. A, B, and C represent the biological replicates.

Sample #	Flow cell #	Sample #	Flow cell #	Sample #	Flow cell #	Sample #	Flow cell #	Barcode #	Barcode sequence
1 /A	1	1 h/A	3	1 /A	5	1 h/A	7	BC01	AAGAAAGTTGTCGGTGTCTTTGTG
1 /B		1 h/B		1 /B		1 h/B		BC02	TCGATTCCGTTTGTAGTCGTCTGT
1 /C		1 h/C		1 /C		1 h/C		BC03	GAGTCTTGTGTCCCAGTTACCAGG
2 /A		1 h/A		2 /A		1 h/A		BC04	TTCGGATTCTATCGTGTTTCCCTA
2 /B		1 h/B		2 /B		1 h/B		BC05	CTTGTCCAGGGTTTGTGTAACCTT
2 /C		1 h/C		2 /C		1 h/C		BC06	TTCTCGCAAAGGCAGAAAGTAGTC
4 /A		2 h/A		4 /A		2 h/A		BC07	GTGTTACCGTGGGAATGAATCCTT
4 /B		2 h/B		4 /B		2 h/B		BC08	TTCAGGGAACAAACCAAGTTACGT
4 /C		2 h/C		4 /C		2 h/C		BC09	AACTAGGCACAGCGAGTCTTGGTT
6 /A		2 h/A		6 /A		2 h/A		BC10	AAGCGTTGAAACCTTTGTCCTCTC
6 /B		2 h/B		6 /B		2 h/B		BC11	GTTTCATCTATCGGAGGGAATGGA
6 /C		2 h/C		6 /C		2 h/C		BC12	CAGGTAGAAAGAAGCAGAATCGGA
8 /A	2	3 h/A	4	8 /A	6	3 h/A	8	BC13	AGAACGACTTCCATACTCGTGTGA
8 /B		3 h/B		8 /B		3 h/B		BC14	AACGAGTCTCTTGGGACCCATAGA
8 /C		3 h/C		8 /C		3 h/C		BC15	AGGTCTACCTCGCTAACACCACTG
1 h/A		4 h/A		1 h/A		4 h/A		BC16	CGTCAACTGACAGTGGTTCGTACT
1 h/B		4 h/B		1 h/B		4 h/B		BC17	ACCCTCCAGGAAAGTACCTCTGAT
1 h/C		4 h/C		1 h/C		4 h/C		BC18	CCAAACCCAACAACCTAGATAGGC
1 h/A		7 h/A		1 h/A		7 h/A		BC19	GTTCCTCGTGCAGTGTCAAGAGAT
1 h/B		7 h/B		1 h/B		7 h/B		BC20	TTGCGTCCTGTTACGAGAACTCAT
1 h/C		7 h/C		1 h/C		7 h/C		BC21	GAGCCTCTCATTGTCCGTTCTCTA
1 h/A		9 h/A		1 h/A		9 h/A		BC22	ACCACTGCCATGTATCAAAGTACG
1 h/B		9 h/B		1 h/B		9 h/B		BC23	CTTACTACCCAGTGAACCTCCTCG
1 h/C		9 h/C		1 h/C		9 h/C		BC24	GCATAGTTCTGCATGATGGGTTAG

## Data summary

The raw sequencing reads were mapped to both the SARS-CoV-2 and to the host reference genomes. In this study, we generated full-length transcripts of SARS-CoV-2 and Vero cells, yielding about 5,462 Gbs of mapped sequencing data. Sequencing of the time-course experiment (dcDNA sequencing) yielded 1,516,913 and 21,246,856 high-quality (Q-score ≥ 8) reads aligned to the viral and the host genome, respectively (Supplementary Tables S1 and S2), while the dRNA sequencing generated 236,518 viral and 1,658,588 *Chlorocebus sabaeus* reads. The ratio of viral transcripts is less than 4% at the first 12 examined time points (1–24 hours pi), and the relative viral read count is the highest at 36 hours pi (Supplementary Table S1, Supplementary Figs. S4, S5). The ratio between the virus–host reads is 14% in the mixed time-point sample (dRNA sequencing). The exact ratio is dependent on the stage of the viral life cycle at the examination period.

The average read length aligning to the SARS-CoV-2 genome was 1,636 bps (it varied from 1,482 to 2,300 bps between the samples) at the time-course dcDNA experiment (Supplementary Table S1). The dRNA-seq resulted in 1,652 bp read length on average.

In accordance with the previously published data [36], our results also show that insertions are the least frequent errors in ONT MinION sequencing (Supplementary Table S1). In agreement with others’ results [37], our dRNA reads have higher deletion and mismatch error rate than the dcDNA-seq samples. In sum, the absolute error rate of the ONT MinION platform is relatively high, which is compensated by the high read coverage. It is important to note that read quality is not essential for transcriptome analysis if well-annotated reference genomes are available.

Our transcriptomic survey yielded a very high read coverage across the viral genome (Fig. 3, Supplementary Fig. S4; detailed information, including quality information, is available in Supplementary Table S1). In our experiment, the ratio of these 2 categories started with about 5–9% in the 1 and 2 hours post infection (hpi) samples and, after a more or less steady growth, peaked at 18 to 20 hpi, with about 25–26%, which indicates an active viral infection phase. The ratio then declined and eventually dropped to roughly the same ratio as in the beginning (4–10%) at 72 and 96 hpi (Fig. 2).

The mapped transcript lengths (without gaps) show that the genomic RNAs tend to be longer than the subgenomic RNAs, both in the cDNA and in the dRNA sequencing libraries (Supplementary Fig. S1). The limitation of LRS approaches is their preference for the short sequences, which leads to the underrepresentation of long RNA molecules compared to the short ones. Despite this shortcoming, these techniques can be used for quantitative analysis by, for example, comparing the amounts of the same RNA molecules at distinct time points of infection.

The genome sequencing reads were used to build the assembled sequence (first Hungarian complete SARS-CoV-2 genome sequence, unpublished). After some testing, we were able to assemble a draft genome with the shasta program, using the 109 reads that were longer than 20,000 bps into 1 contig of length 29,782. This genome draft has overall 30 mutations (compared to the original Wuhan isolate) and consequently 3 frame shifts. Nevertheless, the Nextrain results showed that our isolate (SARS-CoV-2_Hun-1_GenomeDraft_v1) was placed very close to the MT560672.1 genome from the VirStrain output, and both isolates were classified into the clade 20A (EU1) of the virus (Fig. 2, Supplementary Information S1, S2, and S3). This shows the overall robustness of both the *de novo* assembly and the VirStrain method.

## Conclusions and Reuse Potential

The datasets provided in this report allow a time-course look at the full-length transcriptome of SARS-CoV-2 over a 96-hour period of infection, which provides a deeper understanding of the molecular biology of the virus (e.g., transcriptional analysis of subgenomic region, analysis of the dynamics of viral replication, examination of the potential interactions between transcription and replication, as well as to study the potential transcript isoforms of the virus). Our data eliminate the limitations of other SARS-CoV-2 transcriptomic experiments. First, we used a high plaque-forming unit per cell (MOI = 5 pfu/cell) for the infection (other studies typically apply 0.1 pfu); therefore, most cells in the culture became infected, and hence the possibility of a second round of infection is excluded. Additionally, due to the high temporal resolution, our data are also useful to precisely measure the alteration of the gene expression of both the virus and the host cell. Third, we provide mock-infected cells, which were harvested at the same time points as the virally infected cells, which allows the identification of gene-network alterations due to the aging of the cell culture and to analyze the temporal changes of gene expression patterns during the cultivation. Virus–host interactions can also be examined. Furthermore, due to the very long reads and high coverage across the viral genome, assembly of this Hungarian isolate and the analysis of potential genome editing events can be achieved from the data. Moreover, the applied direct RNA and direct cDNA sequencing approaches provide independent methods for the validation of novel transcripts. Finally, this dataset can also be used from various bioinformatics aspects: for example, the data can be further analyzed with or used for the testing of bioinformatic programs, including NanoPack [38], SQANTI3 [39], lra [40], LoRTIA [41, 42], or any other programs for LRS data analysis listed in LONG-READ-TOOLS [43, 44]. Potential template switching artifacts can be tested using the transcript annotator developed by our group [42].

The uploaded binary alignment (BAM) files contain reads already mapped to the SARS-CoV-2 reference genome (MT560672.1), as well as to the host genome (GCA_000 409 795.2) using minimap2.

## Additional Files

**Supplementary Table S1**. Summary statistics of the obtained reads from the infected samples. (A) High-quality reads (Q-score ≥8). (B) Low-quality reads (Q-score <8).

**Supplementary Table S2**. Summary statistics of the obtained reads from the mock-infected samples. (A) High-quality reads (Q-score ≥8). (B) Low-quality reads (Q-score <8).

**Supplementary Table S3**. Detailed information about the concentration of RNA and cDNA samples used for library preparation and sequencing. (A) Concentration of RNA samples. Concentration of SARS-CoV-2–infected and mock-infected RNAs waseasured with Qubit 4.0. The concentrations are in ng/μL. A, B, and C represent the 3 biological replicates. (B) Summary table of the poly(A)+ RNA concentrations. Concentrations of polyadenylated RNAs: from SARS-CoV-2–infected cells and from mock-infected cells in ng/μL. A, B, and C represent the 3 biological replicates. (C) The volume of polyA(+) RNA samples (100 ng) used for cDNA generation. A, B, and C represent the 3 biological replicates. (D) The amount (μL) of RNAs used for preparing a mixture for dRNA sequencing. Agencourt Ampure XP bead was used to get a higher concentration for the mixture (500 ng RNA in 9 μL).

**Supplementary Fig. S1**. Violin plot of mapped region length for genomic, subgenomic, and unclassified viral RNAs.

**Supplementary Fig. S2**. VirStrain result showing the most probable strain present in the reads.

**Supplementary Fig. S3**. Illustration of the ratio between SARS-CoV-2 and host cell read counts throughout the experiment. The viral read count was divided by the host read count for each replicate (group). The means and the standard deviations were also calculated and are shown with a straight line.

**Supplementary Fig. S4**. Polar plot representation of sequencing coverages at the examined time points after viral infection (log_10_ scale).

**Supplementary Fig. S5**. Line graph showing the virus and host read counts throughout the experiment.

giac094_GIGA-D-22-00028_Original_Submission

giac094_GIGA-D-22-00028_Revision_1

giac094_GIGA-D-22-00028_Revision_2

giac094_Response_to_Reviewer_Comments_Original_Submission

giac094_Response_to_Reviewer_Comments_Revision_1

giac094_Reviewer_1_Report_Original_Submission Milad Miladi -- 3/30/2022 Reviewed

giac094_Reviewer_2_Report_Original_Submission George Taiaroa -- 4/15/2022 Reviewed

giac094_Reviewer_2_Report_Revision_1 George Taiaroa -- 8/8/2022 Reviewed

giac094_Supplemental_Files

## Abbreviations

ATCC: American Type Culture Collection; dcDNA: direct cDNA (= nonamplified cDNA); dRNA: direct RNA; FBS: fetal bovine serum; LRS: long-read sequencing; MEM: Minimum Essential Medium Eagle; MOI: multiplicity of infection; PBS: phosphate-buffered saline; pfu: plaque-forming unit; pi: postinfection; RdRP: RNA-dependent RNA polymerase; SARS-CoV-2: severe acute respiratory syndrome coronavirus 2; sgRNA: subgenomic RNA; TRS: transcription-regulating sequence; Vero E6: African green monkey kidney.

## Funding

Supported by National Research, Development and Innovation Office, researcher-initiated research projects, K128247, Z. Boldogkői; National Research, Development and Innovation Office, research projects initiated by young researchers, FK128252, D. Tombácz; Hungarian Academy of Sciences, Momentum Grant, LP2020-8/2020, D. Tombácz; University of Szeged, Open Access Fund, 5654, Z. Boldogkői; and Hungarian Ministry of Innovation and Technology, National Academy of Scientist Education, FEIF/646–4/2021-ITM_SZERZ, Á. Harangozó.

## Data Availability

All data generated in this study, including the unmapped reads as well as reads that do not match our strict criteria (Q-score below 8), can be found in the European Nucleotide Archive under accession number PRJEB51064. Supplementary figures, tables, and information files, including the assembled genome sequence and annotation and the full output of the Nextclade, Virstrain, and Readstatistc programs, as well as other supporting data, be found at the *GigaScience* GigaDB Database [45]. Scripts are available at the GitHub archive [33].

## Competing Interests

The authors declare that there are no conflicts of interest.

## Ethical Approval

Ethical approval for the study was obtained from the institutional review board and research ethic committee of the Complex Medical Center, Budapest, Hungary, under project accession number CMX-U2012. These studies were performed under appropriate containment, given classifications of SARS-CoV-2 at the time of the study.

## Authors' Contributions

D.T. analyzed the data, took part in Nanopore sequencing, drafted the manuscript, and coordinated the project. Á.D. performed Nanopore sequencing and RNA purification. G.G. conducted bioinformatics analysis. Z.C. took part in RNA isolation and sequencing. I.P. participated in RNA isolation and analysis. B.K. carried out bioinformatics. Á.H. participated in sequencing and data analysis. I.J. isolated and propagated the virus. B.D. propagated the virus and the host cells and took part in drafting the manuscript. Z.B. conceived and designed the experiments, supervised the project, and wrote the manuscript. All authors read and approved the final paper.
