# Supplementary material for: High temporal resolution Nanopore sequencing dataset of SARS-CoV-2 and host cell RNAs
Source: Gigascience. 2022 Oct 17;11:giac094. doi: 10.1093/gigascience/giac094 (PMC9575581; doi:10.1093/gigascience/giac094)
Supplement: giac094_GIGA-D-22-00028_Revision_2 [file giac094_giga-d-22-00028_revision_2.pdf]

GigaScience

High Temporal-Resolution Nanopore Sequencing Dataset of SARS-CoV-2 and Host Cell RNAs

--Manuscript Draft--

Manuscript Number:	GIGA-D-22-00028R2
Full Title:	High Temporal-Resolution Nanopore Sequencing Dataset of SARS-CoV-2 and Host Cell RNAs
Article Type:	Data Note
Funding Information:	Innovációs és Technológiai Minisztérium (FEIF/646-4/2021-ITM_SZERZ) — Mr. Ákos Harangozó<br>Nemzeti Kutatási Fejlesztési és Innovációs Hivatal (K 128247) — Prof. Dr. Zsolt Boldogkői<br>Nemzeti Kutatási Fejlesztési és Innovációs Hivatal (FK 128252) — Dr. Dóra Tombácz<br>University of Szeged (Open Access Fund 5654) — Prof. Dr. Zsolt Boldogkői
Abstract:	Background Recent studies have disclosed the genome, transcriptome and epigenetic compositions of severe acute respiratory syndrome coronavirus 2 (SARS-CoV-2) and the effect of viral infection on gene expression of the host cells. It has been demonstrated that, besides the major canonical transcripts, the viral genome also codes for non-canonical RNA molecules. While the structural characterizations have revealed a detailed transcriptomic architecture of the virus, the kinetic studies provided poor and often misleading results on the dynamics of both the viral and host transcripts due to the low temporal resolution of the infection event and the low virus/cell ratio (MOI=0.1) applied for the infection. It has never been tested whether the alteration in the host gene expressions is caused by aging of the cells, or by the viral infection.<br>Findings In this study, we used Oxford Nanopore's direct cDNA and direct RNA sequencing methods for the generation of a high-coverage, high-temporal-resolution transcriptomic dataset of SARS-CoV-2 and of the primate host cells, using a high infection titer (MOI=5). Sixteen sampling time points ranging from 1 to 96 h with a varying time resolution and three biological replicates were used in the experiment. In addition, for each infected sample, corresponding non-infected samples were employed. The raw reads were mapped to the viral and to the host reference genomes, resulting in 49,661,499 mapped reads (54,62Gbs). The genome of the viral isolate was also sequenced and phylogenetically classified.<br>Conclusions This dataset can serve as a valuable resource for profiling the SARS-CoV-2 transcriptome dynamics, the virus-host interactions and the RNA base modifications. Comparison of expression profiles of the host gene in the virally-infected and in non-infected cells at different time points allows to make a distinction between the effect of the aging of cells in culture and the viral infection. These data can provide useful information for potential novel gene annotations and can also be used for studying the currently available bioinformatics pipelines.
Corresponding Author:	Zsolt Boldogkői<br>Szegedi Tudomanyegyetem Altalanos Orvostudomanyi Kar<br>Szeged, HUNGARY
Corresponding Author Secondary Information:	
Corresponding Author's Institution:	Szegedi Tudomanyegyetem Altalanos Orvostudomanyi Kar
Corresponding Author's Secondary Institution:	
First Author:	Dóra Tombácz, PhD
First Author Secondary Information:	
Order of Authors:	Dóra Tombácz, PhD

Powered by Editorial Manager® and ProduXion Manager® from Aries Systems Corporation

	Ákos Dörmő
	Gábor Gulyás
	Zsolt Csabai, PhD
	István Prazsák, PhD
	Balázs Kakuk
	Ákos Harangozó
	István Jankovics, MD
	Béla Dénes, PhD
	Zsolt Boldogkői, PhD
**Order of Authors Secondary Information:**	
**Response to Reviewers:**	Dear Hans,<br>Thank you for your feedback and suggestions.<br>We completed all the necessary corrections in the manuscript, including:<br>- provided reference for the GigaDB dataset<br>- omitted the Source code availability section<br>- removed highlights and tracking<br>- corrected the BioProject ID<br>- corrected references for supporting data<br>- provided numbered citations for every software used<br>We hope now the manuscript is apt for publication.
**Additional Information:**	
**Question**	**Response**
Are you submitting this manuscript to a special series or article collection?	No
**Experimental design and statistics**<br><br>Full details of the experimental design and statistical methods used should be given in the Methods section, as detailed in our Minimum Standards Reporting Checklist. Information essential to interpreting the data presented should be made available in the figure legends.<br><br>Have you included all the information requested in your manuscript?	Yes
**Resources**<br><br>A description of all resources used, including antibodies, cell lines, animals and software tools, with enough information to allow them to be uniquely identified, should be included in the Methods section. Authors are strongly encouraged to cite Research Resource	Yes

Identifiers (RRIDs) for antibodies, model organisms and tools, where possible.<br><br>Have you included the information requested as detailed in our Minimum Standards Reporting Checklist?	
**Availability of data and materials**<br><br>All datasets and code on which the conclusions of the paper rely must be either included in your submission or deposited in publicly available repositories (where available and ethically appropriate), referencing such data using a unique identifier in the references and in the “Availability of Data and Materials” section of your manuscript.<br><br>Have you have met the above requirement as detailed in our Minimum Standards Reporting Checklist?	Yes

Manuscript
Click here to access/download;Manuscript;renamed_ece88.docx

High Temporal-Resolution Nanopore Sequencing Dataset of SARS-CoV-2 and Host Cell RNAs

Dóra Tombácz[1], Ákos Dörmő[1], Gábor Gulyás[1], Zsolt Csabai[1], István Prazsák[1], Balázs Kakuk[1], Ákos Harangozó[1], István Jankovics[2], Béla Dénes[3], Zsolt Boldogkői[1*]

Dóra Tombácz [0000-0001-5520-2978]; Ákos Dörmő [0000-0003-1860-1122]; Gábor Gulyás [0000-0002-7465-7932]; Zsolt Csabai [0000-0003-0031-0116]; István Prazsák [0000-0003-3195-503X]; Balázs Kakuk [0000-0002-4314-5707]; Ákos Harangozó [0000-0001-8499-8485]; István Jankovics [0000-0001-5329-9294]; Béla Dénes [0000-0002-9889-529X]; Zsolt Boldogkői [0000-0003-1184-7293]

[1]Department of Medical Biology, Albert Szent-Györgyi Medical School, University of Szeged, Somogyi B. u. 4., 6720 Szeged, Hungary

[2]Complex Medical Center, 1012 Budapest, Kuny Domonkos u. 13-15, Hungary

[3]Veterinary Diagnostic Directorate, National Food Chain Safety Office, 1143 Budapest, Tábornok u. 2, Hungary

E-Mails:

DT: tombacz.dora@med.u-szeged.hu

ÁD: dormo.akos@med.u-szeged.hu

GG: gulyas.gabor@med.u-szeged.hu

ZC: csabai.zsolt@med.u-szeged.hu

IP: prazsak.istvan@med.u-szeged.hu

BK: kakuk.balazs@med.u-szeged.hu

ÁH: harangozoa5@gmail.com

IJ: jankovics.istvan@nnk.gov.hu

BD: denesb@nebih.gov.hu

ZB: boldogkoi.zsolt@med.u-szeged.hu

*Corresponding author: ZB

Abstract

Background Recent studies have disclosed the genome, transcriptome and epigenetic compositions of severe acute respiratory syndrome coronavirus 2 (SARS-CoV-2) and the effect of viral infection on gene expression of the host cells. It has been demonstrated that, besides the major canonical transcripts, the viral genome also codes for non-canonical RNA molecules. While the structural characterizations have revealed a detailed transcriptomic architecture of the virus, the kinetic studies provided poor and often misleading results on the dynamics of both the viral and host transcripts due to the low temporal resolution of the infection event and the low virus/cell ratio (MOI=0.1) applied for the infection. It has never been tested whether the alteration in the host gene expressions is caused by aging of the cells, or by the viral infection.

Findings In this study, we used Oxford Nanopore's direct cDNA and direct RNA sequencing methods for the generation of a high-coverage, high-temporal-resolution transcriptomic dataset of SARS-CoV-2 and of the primate host cells, using a high infection titer (MOI=5). Sixteen sampling time points ranging from 1 to 96 h with a varying time resolution and three biological replicates were used in the experiment. In addition, for each infected sample, corresponding non-infected samples were employed. The raw reads were mapped to the viral and to the host reference genomes, resulting in 49,661,499 mapped reads (54,62Gbs). The genome of the viral isolate was also sequenced and phylogenetically classified.

Conclusions This dataset can serve as a valuable resource for profiling the SARS-CoV-2 transcriptome dynamics, the virus-host interactions and the RNA base modifications. Comparison of expression profiles of the host gene in the virally-infected and in non-infected cells at different time points allows to make a distinction between the effect of the aging of cells in culture and the viral infection. These data can provide useful information for potential novel gene annotations and can also be used for studying the currently available bioinformatics pipelines.

Keywords: SARS-CoV-2, coronavirus, long-read sequencing, full-length transcriptome, Oxford Nanopore Technologies, MinION system, direct RNA sequencing, direct cDNA sequencing

Data Description

Background

Severe acute respiratory syndrome coronavirus 2 (SARS-CoV-2) is a positive-sense single RNA-stranded betacoronavirus and the etiological agent of the current COVID-19 pandemic [1]. The replication and the transcription of the RNA genome are interrelated because the same enzyme, an RNA-dependent RNA polymerase (RdRP), carries out both processes [2]. First, negative-sense RNA intermediates are generated to serve as templates for the synthesis of both the genomic RNA (gRNA) and the nested set of subgenomic RNAs (sgRNAs) [3]. The gRNA and the sgRNAs have common 5' and 3'-termini since the RdRP synthesizes the positive sense RNAs from this end of the genome. Template-switching occurs during the synthesis of the negative-strand of sgRNAs, which is mediated by the transcription-regulating sequences (TRSs) in the genome body (TRS-B) and in the 5'-leader sequence (TRS-L) resulting in the fusion of leader-body sequences [4,5]. Recent studies have disclosed the transcriptomic architecture of SARS-CoV-2 and the effect of viral infection on the host gene expression [6]. It has been shown that, besides canonical TRS-dependent RNA molecules, the viral genome also codes for non-canonical, TRS-dependent and TRS-independent, transcripts, although in a relative low abundance (altogether <10%). Additionally, investigations of the effect of the viral

infection on the transcriptome of various cell types have identified several genes and gene networks [7].

Nonetheless, the kinetic studies of gene expressions used only a few timepoints for monitoring the infection [8,9], which do not provide a comprehensive picture on the temporal dynamics of viral transcriptome. Furthermore, typically a low (0.1) multiplicity of infection (MOI) was applied in the experiments, which may lead to misleading conclusions on the kinetic properties of SARS-CoV-2 transcripts, because after the completion of the replication cycle, the virus can gradually initiate new infection cycles within the non-infected cells [10]. Low-MOI-infection makes also difficult to assess the host cell response, especially in the case of the down-regulated genes. Infections are typically carried out using fresh, rapidly growing cultured cells, however, only the fresh cells (at time point 0) are used as mock-infected cells. Nonetheless, gene expression profiles may undergo alterations in non-infected cells during the propagation therefore, we cannot decide whether the transcriptional changes in infected cells are due to the effect of the virus or to the time factor of culturing (aging of cells). This phenomenon has practically never been tested in the experiments. An additional problem is the use of short-read sequencing for profiling of the host cell reaction to the viral infection [7] because this approach has severe limitations for the detection of transcript isoforms, such as splice and length variants, and multigenic transcripts, among others [11-13].

Long-read sequencing (LRS) opened new avenues for the comprehensive analysis of the transcriptomes, for which the major reason is that these techniques are able to detect full-length RNA molecules and thereby to distinguish between transcript isoforms and transcriptional overlaps. LRS-based studies have revealed a hidden transcriptional complexity in viruses [14-17], but this approach has also been used for the analysis of the kinetic properties of viral transcriptomes [18], for the analysis of RNA modifications [16,19], as well as the virus-host interaction [20,21].

In this study, we applied nanopore sequencing based on direct RNA (dRNA) and direct cDNA (dcDNA) approaches for the generation of transcriptomic datasets from SARS-CoV-2 and primate host

Vero) cells. A mixed time point sample (single library from a mixture containing equal amount of total RNAs from each of the 16 time points) was used for dRNA sequencing, while we used 16 time-point samples within an interval of 1 to 96 h from both infected and non-infected host cells using MOI=5 for the infection.

Decoding the transcriptional landscape of SARS-CoV-2 virus is a fundamental step in studying its biology, genetic regulation and molecular pathogenesis. Therefore, in this data descriptor, our aim was to provide a robust, precise, reliable dataset based on LRS approaches for understanding the gene expression and genetic regulation of the causative agent of current pandemic, its effect on differential host gene expression, as well as to provide a rich resource for future functional studies.

Table 1.

	**Quality**	**Total (infected)**	**Virus**	**Host (infected)**	**Unmapped (infected)**	**Total (uninfected)**	**Host (uninfected)**	**Unmapped (uninfected)**
**dcDNA**	**all**	**32,017,113**	**1,527,249**	**23,703,827**	**6,786,037**	**29,294,533**	**22,149,844**	**7,144,689**
**dcDNA**	≥8	23,607,200	1,280,395	21,246,856	1,079,949	20,360,096	19,008,016	1,352,080
**dcDNA**	<8	8,409,913	246,854	2,456,971	5,706,088	8,934,437	3,141,828	5,792,609
**dRNA**	**all**	**2,606,502**	**281,418**	**1,999,161**	**325,923**	-	-	-
**dRNA**	≥8	1,950,595	236,518	1,658,588	55,489	-	-	-
**dRNA**	<8	655,907	44,900	340,573	270,434	-	-	-

Methods

Figure 1 shows the detailed workflow of the study.

Cells

The Vero E6 (African green monkey kidney) cell line was obtained from the American Type Culture Collection (ATCC). The cells were plated at a density of 2 x 10^6 cells per 75 cm^2 tissue culture flasks

(CELLSTAR® Greiner Bio-One GmbH, Frickenhausen, Germany) in Minimum Essential Medium Eagle culture medium (MEM) with 10% fetal bovine serum (FBS) and 2mM L-glutamine and antibiotic-antimycotic solution (all obtained from Sigma-Aldrich). Vero cells were incubated at 37°C in a humidified 5% CO_2 atmosphere until confluency (~8 x 10^6 cells) was reached. The monolayer was washed once with the serum-free MEM immediately before infection.

Collection, detection and isolation of the virus

The SARS-CoV-2 virus was isolated from the human nasopharyngeal swab of the RT-PCR positive (Ct 22) 77-year-old male patient during the official COVID-19 surveillance program at the Veterinary Diagnostic Directorate of the National Food Chain Safety Office (Budapest, Hungary) with the cooperation of the Complex Medical Center (Budapest) in November 2020 at the second wave of COVID-19 pandemic in Hungary. The patient developed respiratory illness, with fever, cough, and fatigue that quickly progress to pneumonia. The patient was hospitalized, where, unfortunately, he died in a few days. In his story, he did not declare any travel abroad in the last 14 days. At the same time, he traveled relatively frequently within Hungary and had been in close contact with the people with COVID-19.

Detection of SARS-CoV-2 in pharyngeal wash samples was performed using RT-PCR amplification of SARS-CoV-2 N-gene fragments. Two hundred microliters (200 µL) of the pharyngeal washes were first processed for RNA extraction in the Thermo Scientific™ KingFisher™ Flex Purification System (Thermo Fisher Scientific, Waltham, MA USA), using the IndiMag® Pathogen Kit (QIAGEN® GmbH, Hilden, Germany). Subsequently, the detection of N-gene of SARS-CoV-2 was performed by using the 2019-nCoV-2 RUO kit (Integrated DNA Technologies, Inc., Coralville, Iowa, USA) and One-Step RT-PCR Kit (QIAGEN® GmbH) on a Rotor-Gene Q real-time PCR cycler (QIAGEN® GmbH). The amplification protocol consisted of a reverse transcription step at 50°C for 30 minutes, a denaturation step at 95°C for 15 minutes and subsequent 45 cycles at 95°C/56°C/72°C for 30/30/60

seconds, respectively. A positive result was defined as amplification of N-gene in a sample with each cycle threshold value (ct) less than 37.

For the virus isolation, 1 ml of viral transport media from the swab was mixed with 3 ml serum-free MEM culture medium supplemented with 2mM L-glutamine and antibiotic-antimycotic solution and were filtered using Ministar® 0.22µm filter (Sartorius AG, Göttingen, Germany). The filtrate was placed onto cells in a 25 cm^2 tissue culture flask (Corning®, Corning Inc., New York, USA) of Vero E6 cells, then were incubated at 37°C in a humidified 5% CO_2 chamber for 1 hour. After incubation, two and a half milliliters (2.5 ml) of serum-free MEM culture medium with 2mM L-glutamine and antibiotic-antimycotic solution was added to the tissue culture flask. The inoculated culture was grown in a humidified 37°C incubator with 5% CO_2. Cells were observed daily for cytopathic effect (CPE). On day 4, an 80% CPE was evident, and the cells with supernatant were harvested. This provided the first passage virus. The virus was passaged twice at low MOI in Vero E6 cells to obtain a working stock used in the experiments. The viral titer was determined by plaque assay on Vero E6 cells. Virus stock was stored at -80°C until use.

Propagation of the virus

The virus was passaged twice in Vero cells to obtain a working stock used in all experiments. Viral titer was determined by plaque assay on Vero cells. The virus was diluted into a serum-free MEM. Cells were infected with 5 ml of the SARS-CoV-2 virus with 5 plaque-forming unit (pfu)/cell [multiplicity of infection (MOI=5)], then were incubated at 4°C for 1 h. Non-infected control cultures (mock) were prepared using pure non-supplemented MEM as inoculums. Next, the virus inoculum was removed from the flasks. The monolayer was washed once with the serum-free MEM. Ten milliliters of MEM culture medium supplemented with 3% FBS, 2mM L-glutamine, and antibiotic-antimycotic solution was added to the tissue culture flasks. The cells were incubated at 37°C for 1, 2, 4, 6, 8, 10, 12, 14, 16, 18, 20, 24, 36, 48, 72 and 96 hours in a humidified 5% CO_2 atmosphere. Each time experiment was done in triplicate with a mock-infected control. Mock-infected cells were harvested at

the same time points as the infected cells. Following incubation, the medium was removed, and the monolayer was washed once with phosphate-buffered saline (PBS). The tissue culture plates were stored at -80˚C until use. Next, the infected cells were treated by lysis buffer, then creped and placed into an Eppendorf Tubes® (Thermo Fisher Scientific Inc.).

RNA purification

Total RNA was extracted from the mock-infected and from the SARS-CoV-2-infected cells at various stages of infection from 1 to 96 hours using the Macherey-Nagel's NucleoSpin RNA Kit according to the manufacturer's protocol. In brief, cells were collected by low-speed centrifugation, then 350µl lysis buffer (RA1 from the Kit), 3.5µl β-Mercapthoethanol (Sigma Aldrich) were added followed by vortexing the samples. Mixtures were loaded onto a NucleoSpin Filter and centrifuged for 1min at 11,000 x g. The filters were discarded and 350µl 70% EtOH was added to the lysate. This was loaded to the NucleoSpin RNA Column and centrifuged at 11,000 x g for 30sec. Membrane was desalted with the addition of 350µl Membrane Desalting Buffer (from the NucleoSpin Kit), then dried with a short centrifugation (11,000 x g). Residual DNA was enzymatically removed [with the usage of 95µl mixture of rDNase:rDNase reaction buffer (1:9 ratio, NucleoSpin Kit) and incubation at room temperature (RT) for 15min]. The rDNase was inactivated with the first washing step, by adding 200µl RAW2 Buffer (NucleoSpin Kit) directly onto the NucleoSpin Filter. After a quick centrifugation (30min, 11,000 x g), the filter was placed in a new tube. Six-hundred µl RAW3 Buffer (NucleoSpin Kit) was added, then spun down as before. This washing step was repeated using 250µl RAW3. Finally, the total RNA bound to the Filter was eluted in 60µl nuclease-free water (NucleoSpin Kit). Samples were quantified by Qubit 4.0 using the Qubit RNA BR Assay Kit (Invitrogen, **Supplementary Table S3A**) and then stored at -80°C until use.

Poly(A) selection

Lexogen's Poly(A) RNA Selection Kit V1.5 was used to isolate polyadenylated RNAs from the total RNA samples. The protocol applies oligo(dT) beads, which capture RNAs with poly(A) stretches (most

mRNAs), but RNAs without polyadenylated 3′ ends (e.g. 28S and 18S rRNAs and tRNAs) do not hybridize to the beads and therefore, they will be removed during the washing steps. The detailed protocol is as follows: the magnetic beads (part of the Lexogen Kit) was resuspended and 4µl for each RNA samples was measured. Beads were placed in a magnet, they were collected and the supernatant was discarded. Samples were resuspended in 75µl Bead Wash Buffer (Lexogen Kit) and then were placed on the magnet. Supernatant was discarded and this washing step was repeated once. Beads were resuspended in 20µl RNA Hybridization Buffer (part of the Lexogen Kit). Ten ng from the total RNA samples were diluted to 20µl UltraPure™ DNase/RNase-Free Distilled Water (Invitrogen) and then denatured at 60°C for 1min followed by holding them at 25°C. Twenty µl denatured RNA was mixed with 20µl (previously washed and resuspended) beads. The mixtures were incubated at 25°C in a shaker incubator with 1250 rpm agitation. After 20min incubation, sample-containing tubes were placed in a magnetic rack. Supernatant was discarded then the tubes were removed from the magnet. Samples were resuspended in 100µl Bead Wash Buffer (Lexogen Kit) then they were incubated for 5min at 25°C with 1250 rpm agitation. Supernatant was discarded and the washing step was repeated. After the complete removal of the supernatant, beads were resuspended in 12µl UltraPure™ DNase/RNase-Free Distilled Water. Samples were incubated at 70°C for 1min, then the tubes were placed on a magnetic rack. Supernatant, containing the poly(A)+ RNA fraction, was placed to new DNA LoBind (Eppendorf) tubes, the RNA concentration was measured using Qubit RNA HS Assay Kit (Invitrogen, **Supplementary Table S3B**), then samples were stored at -80°C.

ONT – direct cDNA sequencing

For the analysis of the dynamic properties of SARS-CoV-2 RNAs and the effect of viral infection on the host cell transcriptome profile, RNA samples from different time points (1, 2, 4, 6, 8, 10, 12, 14, 16, 18, 20, 24, 36, 48, 72 and 96h p.i., **Supplementary Table S3C**) were used individually for the generation of direct cDNA libraries for nanopore sequencing. The non-amplified cDNA libraries were prepared from sixteen time-points from the mock, and from the coronavirus-infected samples, in three

biological replicates using the Direct cDNA Sequencing Kit (SQK-DCS109, ONT) and the appropriate ONT protocol. In short, first-strand cDNAs were generated from the polyA(+) RNAs using the Maxima H Minus Reverse Transcriptase (Thermo Fisher Scientific) with SSP and VN primers (supplied in the kit). The RNase Cocktail Enzyme Mix (Thermo Fisher Scientific) was used to eliminate the potential RNA contamination. Synthesis of the second cDNA strands were carried out with LongAmp Taq Master Mix (New England Biolabs). The double-stranded cDNAs were repaired (NEBNext End repair /dA-tailing Module, New England Biolabs) and adapter ligated (NEB Blunt /TA Ligase Master Mix New England Biolabs). Individual barcode sequences were added to each samples for multiplex sequencing for which the Native Barcoding (12) Kit (ONT) was used as recommended by the manufacturer. The cDNAs and the libraries were washed using AMPure XP beads (Agencourt, Beckman Coulter) after every enzymatic reaction step. The barcode labeled samples were loaded onto MinION R9.4 SpotON Flow Cells (ONT, **Table 2**).

Table 2.

								Barcode #	Barcode sequence
**Sample #**	**Flow cell #**	**Sample #**	**Flow cell #**	**Sample #**	**Flow cell #**	**Sample #**	**Flow cell #**		
1h/A	1	16h/A	3	1h/A	5	16h/A	7	BC01	AAGAAAGTTGTCGGTGTCTTTGTG
1h/B		16h/B		1h/B		16h/B		BC02	TCGATTCCGTTTGTAGTCGTCTGT
1h/C		16h/C		1h/C		16h/C		BC03	GAGTCTTGTGTCCCAGTTACCAGG
2h/A		18h/A		2h/A		18h/A		BC04	TTCGGATTCTATCGTGTTTCCCTA
2h/B		18h/B		2h/B		18h/B		BC05	CTTGTCCAGGGTTTGTGTAACCTT
2h/C		18h/C		2h/C		18h/C		BC06	TTCTCGCAAAGGCAGAAAGTAGTC
4h/A		20h/A		4h/A		20h/A		BC07	GTGTTACCGTGGGAATGAATCCTT
4h/B		20h/B		4h/B		20h/B		BC08	TTCAGGGAACAAACCAAGTTACGT
4h/C		20h/C		4h/C		20h/C		BC09	AACTAGGCACAGCGAGTCTTGGTT
6h/A		24h/A		6h/A		24h/A		BC10	AAGCGTTGAAACCTTTGTCCTCTC
6h/B		24h/B		6h/B		24h/B		BC11	GTTTCATCTATCGGAGGGAATGGA
6h/C		24h/C		6h/C		24h/C		BC12	CAGGTAGAAAGAAGCAGAATCGGA
8h/A	2	36h/A	4	8h/A	6	36h/A	8	BC13	AGAACGACTTCCATACTCGTGTGA
8h/B		36h/B		8h/B		36h/B		BC14	AACGAGTCTCTTGGGACCCATAGA
8h/C		36h/C		8h/C		36h/C		BC15	AGGTCTACCTCGCTAACACCACTG
10h/A		48h/A		10h/A		48h/A		BC16	CGTCAACTGACAGTGGTTCGTACT
10h/B		48h/B		10h/B		48h/B		BC17	ACCCTCCAGGAAAGTACCTCTGAT
10h/C		48h/C		10h/C		48h/C		BC18	CCAAACCCAACAACCTAGATAGGC
12h/A		72h/A		12h/A		72h/A		BC19	GTTCCTCGTGCAGTGTCAAGAGAT
12h/B		72h/B		12h/B		72h/B		BC20	TTGCGTCCTGTTACGAGAACTCAT
12h/C		72h/C		12h/C		72h/C		BC21	GAGCCTCTCATTGTCCGTTCTCTA
14h/A		96h/A		14h/A		96h/A		BC22	ACCACTGCCATGTATCAAAGTACG
14h/B		96h/B		14h/B		96h/B		BC23	CTTACTACCCAGTGAACCTCCTCG
14h/C		96h/C		14h/C		96h/C		BC24	GCATAGTTCTGCATGATGGGTTAG

ONT – direct RNA sequencing

ONT's Direct RNA sequencing (SQK-RNA002; Version: DRS_9080_v2_revO_14Aug2019, Last update: 10/06/2021) was used to sequence the native RNA strands from a mixture of polyA(+) RNA fractions (**Supplementary Table S3D**). Five-hundred ng RNA in 9µl nuclease-free water was mixed with 3µl NEBNext Quick Ligation Reaction Buffer (New England BioLabs), 0.5µl RNA CS (ONT Kit), 1µl RT Adapter (110nM; ONT Kit) and 1.5µl T4 DNA Ligase (2M U/ml New England BioLabs). The ligation reaction was carried out for 10min at RT. The synthesis of the first strand cDNA was conducted using SuperScript III Reverse Transcriptase (Life Technologies), as described in the Direct RNA sequencing (DRS) protocol (ONT). In short, a 50 min incubation at 50°C was followed by the inactivation of the enzyme at 70°C for 10 min. Sequencing adapters from the DRS kit were ligated to the cDNA with the T4 DNA ligase enzyme and NEBNext Quick Ligation Reaction Buffer (New England BioLabs). Ligation was carried out at RT for 10 min. The sample was washed using AMPure XP beads (Agencourt, Beckman Coulter) after every enzymatic reaction. Libraries were sequenced on an R9.4 SpotON Flow Cell.

Technical Validation

RNA The Qubit RNA BR Assay Kit (Invitrogen, Carlsbad, CA, United States) was used to check the amount of total RNA. Qubit RNA HS Assay Kit (Invitrogen, Carlsbad, CA, United States) was used to measure the quantity of the poly(A)+ RNA fractions. The final concentrations of the RNA samples were determined by Qubit® 4.

cDNA The concentrations of the cDNA samples and sequencing ready libraries were measured using the Qubit dsDNA HS Assay Kit (Invitrogen, Carlsbad, CA, United States). The quality of RNA was assessed using the Agilent 2200 TapeStation System. RIN scores ≥ 9.6 were used for sequencing (**Figure 1D**).

The cDNAs and the sequencing-ready cDNA libraries were washed using AMPure XP beads (Agencourt, Beckman Coulter) after every enzymatic reaction. The samples for dRNA sequencing were treated with RNAClean XP beads.

Three biological replicates were used for each of the 16 time points. To monitor the effect of SARS-CoV-2 infection on the gene expression of the host cells, mock-infected cells were harvested at the same time-points, as the virally-infected cells.

Data analysis

The MinION raw data was basecalled using ONT Guppy basecalling software version 5.0.11. using --qscore_filtering: reads with a Q-score of 8 or greater were termed as '*passed'* and those below were termed as '*failed'*. The VirStrain [22] tool was used on the 'passed' reads to identify the closest SARS-CoV-2 strains to our isolate (**Supplementary Figure S1** and **Supplementary Info S1**). The resulting most likely genome (NCBI nucleotide accession: MT560672.1) was used as reference for the mapping of the reads. The infected samples reads were mapped to the host (*Chlorocebus_sabeus* 1.1) genome (GenBank assembly accession: GCA_000409795.2) as well, while the mock (uninfected) samples were mapped to the host genome only. The mappings were carried out with the *minimap2* aligner [23], using the following parameters: *minimap2 -ax splice -Y -C5*. The *view* command from the SamTools package [24] was used on the resulting 'sam' files to generate binary alignment ('.bam') files, which were subsequently sorted and indexed, using the *sort* and *index* commands, respectively; and finally the *view* command was used again to separate the data into viral-mapped, host-mapped and unmapped '.bam' files. Our in-house developed python script 'readstats.py' were used to generate the descriptive statistics of reads and the alignments [25]. The output of *readstat* script, containing the mapping statistics was imported into R. Subsequently the median, 25% percentile and 75% percentile values of the mapped read lengths were calculated and visualized using ggplot2 [26] for both the viral and host reads (**Figure 5**). In the case of the infected samples, the ratios of the reads mapped to the viral and host genome were also visualized using ggplot2 (**Supplementary Figure S3**).

To distinguish RNAs originating from the viral genome (gRNA) from the sub-genomic transcripts (sgRNA), we further processed the reads by re-mapping the reads initially mapped to the original Wuhan genome isolate (NC_145512.2) with *minimap2 -ax splice -Y -C5 --MD -un -g 30000 -G 30000*

-O2,24 -E1,0 -C0 -z 400,200 --no-end-flt -F 40000 --secondary=no --splice-flank=no --max-chain-skip=40 --for-only. The alignments were subsequently imported into *R* and processed via an in-house developed script, utilizing packages of the tidyverse [27], RSamtools [28], GenomicAlignments [29], tidygenomics [30] and dplyr [31]. Sub-genomic RNAs were defined as RNAs that overlap with either sub-genomic ORF and have a template switch, connecting this mapped region with the 5'-leader part of the genome (in the 55-85 position of the reference genome). Genomic RNAs were defined as those RNAs that overlap with ORF1ab (with at least 10 nt-s), and are not in the sub-genomic category. All other reads were categorized as 'unclassified' RNAs. The ratio of the sub-genomic/genomic categories in each sample was visualized in a scatterplot with a fitted loess function (**Figure 2**).

From the imported alignments, genome coverage was calculated and subsequently visualized in a log10 scale (**Figure 4**) using ggplot2 [26], the ORF annotations was generated with *gggenes* [32].

The mapped parts of the RNAs were summed to calculate transcript lengths. From this data, violin plots were generated for the genomic, sub-genomic and unclassified RNAs as well (**Supplementary Figure S1**).

The scripts that were used to analyze the alignments and to classify them as genomic or sub-genomic origin, is available as a complete workflow, that is, from downloading the reads to generate the figures, at a gtihub repository [33]. The R-scripts can be used with other *bam* files, reference genomes and/or parameters, as well to import, filter and analyze alignments or to dereplicate them into transcripts.

The SARS-CoV-2 genome was assembled with the shasta program (v.0.9.0) [34] using all viral reads longer than 20,000 bps (*shasta --Reads.minReadLength 20000 --config Nanopore-Oct2021*; otherwise default parameters). The obtained draft assembly (SARS-CoV-2_Hun-1_GenomeDraft_v1) was analyzed for mutations and characterized phylogenetically with the Nextstrain [35] program, along with the genome from the VirStrain result (**Figure 3, Supplementary Figure S2, Supplementary Info S1, S2, S3 and Supplementary Table S4**. The draft assembly was submitted to NCBI (sequence accession: OM812693.1).

Altogether, we generated almost 64 million long-reads from which more than 1.8 million reads mapped to the SARS-CoV-2 and almost 48 million to the host reference genome (**Table 1)**. Time course changes in the virus to host ratio is depicted in **Supplementary Figure S3**. The obtained read count resulted in a very high coverage across the viral genome **(Figure 4)**. Detailed data on the read counts, quality of reads including read lengths **(Figure 5)**, insertions, deletions, as well as mismatches are summarized in **Supplementary Table S1A, B and S2A B**.

Data summary

The raw sequencing reads were mapped to both the SARS-CoV-2 and to the host reference genomes. In this study, we generated full-length transcripts of SARS-CoV-2 and the Vero cells, yielding about 54,62 Gbs of mapped sequencing data. Sequencing of the time-course experiment (dcDNA sequencing) yielded 1,516,913 and 21,246,856 high quality (Q-score ≥ 8) reads aligned to the viral and the host genome, respectively (**Supplementary Table S1 and S2)**, while the dRNA sequencing generated 236,518 viral and 1,658,588 *C. sabaeus* reads. The ratio of viral transcripts is less than 4% at the first twelve examined time points (1-24h p.i.), and the relative viral read count is the highest at 36h pi (**Supplementary Table S1, Supplementary Figure S4, S5**). The ratio between the virus-host reads is 14% in the mixed time point sample (dRNA sequencing). The exact ratio is dependent on the stage of the viral life cycle at the examination period.

The average read lengths aligning to the SARS-CoV-2 genome was 1,636 bps (it varies from 1,482 bps to 2,300 bps between the samples) at the time-course dcDNA experiment (**Supplementary Table S1**). The dRNA-seq resulted in 1,652 bp read length in an average.

In accordance with the previously published data [36], our results also show that insertions are the least frequent errors in ONT MinION sequencing (**Supplementary Table S1**). In agreement with others' results [37], our dRNA reads have higher deletion and mismatch error rate than the dcDNA-Seq samples. In sum, the absolute error rate of ONT MinION platform is relative high, which is

compensated by the high read coverage. It is important to note that read quality is not essential for transcriptome analysis if well-annotated reference genomes are available.

Our transcriptomic survey yielded a very high read-coverage across the viral genome (**Figure 3, Supplementary Figure S4,** detailed information, including quality information are available in **Supplementary Table S1**). In our experiment, the ratio of these two categories started with about 5-9% in the 1 and 2 hpi samples, and after a more or less steady growth, peaked at 18-20 hpi, with about 25-26%, which indicates an active viral infection phase. The ratio then declined and eventually dropped to roughly the same ratio as in the beginning (4-10%) at 72 and 96 hpi (**Figure 2**).

The mapped transcript lengths (without gaps) show that the genomic RNAs tend to be longer than the sub-genomic RNAs, both in the cDNA and in the dRNA sequencing libraries (**Supplementary Figure S1**). The limitation of LRS approaches is their preference for the short sequences, which leads to the underrepresentation of long RNA molecules compared to the short ones. Despite this shortcoming, these techniques can be used for quantitative analysis by e.g. comparing the amounts of the same RNA molecules at distinct time points of infection.

The genome sequencing reads were used to build the assembled sequence (first Hungarian complete SARS-CoV-2 genome sequence, unpublished). After some testing, we were able to assemble a draft genome with the shasta program, using the 109 reads that were longer than 20,000 bps into one contig of length 29,782. This genome draft has an overall of 30 mutations (compared to the original Wuhan isolate), and consequently 3 frame-shifts. Nevertheless, the Nextrain results showed that our isolate (SARS-CoV-2_Hun-1_GenomeDraft_v1) was placed very close to MT560672.1 genome from the VirStrain output, both isolates were classified into the clade 20A (EU1) of the virus (**Figure 2, Supplementary Info S1, S2 and S3**). This shows the overall robustness of both the *de novo* assembly and the VirStraind method.

Conclusions and Reuse Potential

The datasets provided in this report allow a time-course look at the full-length transcriptome of SARS-CoV-2 over a 96 h period of infection which provides a deeper understanding of the molecular biology of the virus (e.g. transcriptional analysis of subgenomic region, analysis of the dynamics of viral replication, examination of the potential interactions between transcription and replication, as well as to study the potential transcript isoforms of the virus). Our data eliminate the limitations of other SARS-CoV-2 transcriptomic experiments. First, we used a high plaque-forming unit per cell (MOI=5 pfu/cell) for the infection (other studies typically apply 0.1 pfu), therefore the large majority of cells in the culture became infected, and hence the possibility of a second round of infection is excluded. Additionally, due to the high temporal resolution, our data is also useful to precisely measure the alteration of the gene expression of both the virus and the host cell. Third, we provide mock-infected cells, which were harvested in the same time-points, as the virally-infected cells, which allows the identification of gene-network alterations due to the aging of the cell culture and to analyze the temporal changes of gene expression patterns during the cultivation. Virus-host interactions can also be examined. Furthermore, due to the very long reads and high coverage across the viral genome, assembly of this Hungarian isolate and the analysis of potential genome editing events can be achieved from the data. Moreover, the applied direct RNA and direct cDNA sequencing approaches provides independent methods for the validation of novel transcripts. Finally, this dataset can also be used from various bioinformatics aspects: e.g. the data can be further analyzed with or used for the testing of bioinformatic programs, including NanoPack [38], SQANTI3 [39], lra [40], LoRTIA [41,42] or any other programs for LRS data analysis listed in LONG-READ-TOOLS [43, 44]. Potential template switching artefacts can be tested using the transcript annotator developed by our group [42].

The uploaded binary alignment (BAM) files contain reads already mapped to the SARS-CoV-2 reference genome (MT560672.1), as well as to the host genome (GCA_000409795.2) using minimap2.

Availability of Supporting Data

All data generated in this study including the unmapped reads as well as reads which do not match our strict criteria (Q-score below 8) can be found in European Nucleotide Archive under the accession number: PRJEB51064. Supplementary Figures, Tables and Info files, including the assembled genome sequence and annotation and the full output of the Nextclade, Virstrain and Readstatistc programs; and other supporting data be found at the GigaScience Database [45]. Scripts are available at the github archive [33].

Competing interests

The authors declare that there are no conflicts of interest.

Funding

National Research, Development and Innovation Office, Researcher-initiated research projects, K 128247, Z Boldogkői;

National Research, Development and Innovation Office, Research projects initiated by young researchers, FK 128252, D Tombácz;

Hungarian Academy of Sciences, Momentum Grant, LP2020-8/2020, D Tombácz;

University of Szeged, Open Access Fund, 5654, Z Boldogkői;

Hungarian Ministry of Innovation and Technology, National Academy of Scientist Education, FEIF/646-4/2021-ITM_SZERZ, Á. Harangozó;

Author Contributions

DT analyzed the data, took part in Nanopore sequencing, drafted the manuscript and coordinated the project. ÁD performed Nanopore sequencing and RNA purification. GG conducted bioinformatics analysis. ZC took part in RNA isolation and sequencing. IP participated in RNA isolation and analysis. BK carried out bioinformatics. ÁH participated in sequencing and data analysis. IJ isolated and

propagated the virus. BD propagated the virus and the host cells and took part in drafting the manuscript. ZB conceived and designed the experiments, supervised the project and wrote the manuscript. All authors read and approved the final paper.

Ethical approval

The ethical approval for the study was obtained from the institutional review board and research ethic committee of the Complex Medical Center, Budapest, Hungary, under the project accession number: CMX-U2012. These studies were performed under appropriate containment, given classifications of SARS-CoV-2 at the time of the study.

Abbreviations

ATCC - American Type Culture Collection

dcDNA - direct cDNA (= non-amplified cDNA)

dRNA - direct RNA

FBS - fetal bovine serum

LRS - Long-read sequencing

MEM - Minimum Essential Medium Eagle

MOI - multiplicity of infection

PBS - phosphate-buffered saline

pfu – plaque-forming unit

pi - post-infection

RdRP - RNA-dependent RNA polymerase

SARS-CoV-2 - Severe acute respiratory syndrome coronavirus 2

sgRNA - subgenomic RNA

TRS - transcription-regulating sequence

Vero E6 - African green monkey kidney

References

1. Zhou P, Yang X-L, Wang X-G, Hu B, Zhang L, Zhang W, et al. A pneumonia outbreak associated with a new coronavirus of probable bat origin. *Nature* 2020;579:270-273. doi:10.1038/s41586-020-2012-7

2. Woo PC, Huang Y, Lau SK, Yuen KY. Coronavirus genomics and bioinformatics analysis. *Viruses* 2010;2:1804–20. doi:10.3390/v2081803.

3. Sola I, Moreno JL, Zúñiga S, Alonso S, Enjuanes L. Role of nucleotides immediately flanking the transcription-regulating sequence core in coronavirus subgenomic mRNA synthesis. *J. Virol*. 2005;79:2506-2516. doi:10.1128/JVI.79.4.2506-2516.2005

4. Hussain S, Pan J, Chen Y, Yang Y, Xu J, Peng Y, et al. Identification of novel subgenomic RNAs and noncanonical transcription initiation signals of severe acute respiratory syndrome coronavirus. *J. Virol*. 2005;79:5288-5295. doi:10.1128/JVI.79.9.5288-5295.2005

5. Sola I, Almazán F, Zúñiga S, Enjuanes L. Continuous and Discontinuous RNA Synthesis in Coronaviruses. *Annu. Rev. Virol*. 2015;2:265-288. doi: 10.1146/annurev-virology-100114-055218

6. Kim D, Lee J-Y, Yang J-S, Kim JW, Kim VN, Chang H. The Architecture of SARS-CoV-2 Transcriptome. *Cell* 2020;181:914-921.e10. doi:10.1016/j.cell.2020.04.011

7. Sun J, Ye F, Wu A, Yang R, Pan M, Sheng J, et al. Comparative Transcriptome Analysis Reveals the Intensive Early Stage Responses of Host Cells to SARS-CoV-2 Infection. *Front. Microbiol*. 2020;11:593857. doi:10.3389/fmicb.2020.593857

8. Chang JJ-Y, Rawlinson D, Pitt ME, Taiaroa G, Gleeson J, Zhou C, et al. Transcriptional and epi-transcriptional dynamics of SARS-CoV-2 during cellular infection. *Cell Rep*. 2021;35109108. doi:10.1016/j.celrep.2021.109108

9. Wang D, Jiang A, Feng J, Li G, Guo D, Sajid M, et al. The SARS-CoV-2 subgenome landscape and its novel regulatory features. *Mol. Cell* 2021;81:2135–2147. doi:10.1016/j.molcel.2021.02.036

10. Beyleveld G, White KM, Ayllon J, Shaw ML. New-generation screening assays for the detection of anti-influenza compounds targeting viral and host functions. *Antiviral Res*. 2013;100:120-32. doi:10.1016/j.antiviral.2013.07.018

11. Steijger T, Abril JF, Engström PG, Kokocinski F, The RGASP Consortium, Hubbard TJ, et al. Assessment of transcript reconstruction methods for RNA-seq. *Nat. Methods* 2013;10:1177–1184. doi:10.1038/nmeth.2714

12. Tombácz D, Csabai Z, Oláh P, Balázs Z, Likó I, Zsigmond L, et al. Full-length isoform sequencing reveals novel transcripts and substantial transcriptional overlaps in a herpesvirus. *Plos One* 2016;11:e0162868. doi:10.1371/journal.pone.0162868

13. Prazsák I, Moldován N, Balázs Z, Tombácz D, Megyeri K, Szűcs A, et al. Long-read Sequencing Uncovers a Complex Transcriptome Topology in Varicella Zoster Virus. *BMC Genomics* 2018;19:873. doi:10.1186/s12864-018-5267-8

14. Boldogkői Z, Moldován N, Balázs Z, Snyder M, Tombácz D. Long-read sequencing – a powerful tool in viral transcriptome research. *Trends Microbiol*. 2019;27:578-592. doi:10.1016/j.tim.2019.01.010

15. Tombácz D, Torma G, Gulyás G, Moldován N, Snyder M, Boldogkői Z. Meta-analytic Approach for Transcriptome Profiling of Herpes Simplex Virus Type 1. *Sci. Data*, 2020;7: 223. doi:10.1038/s41597-020-0558-8

16. Viehweger A, Krautwurst S, Lamkiewicz K, Madhugiri R, Ziebuhr J, Hölzer M, et al. Direct RNA nanopore sequencing of full-length coronavirus genomes provides novel insights into structural variants and enables modification analysis. *Genome Res*. 2019;29:1545-1554. doi:10.1101/gr.247064.118

17. Taiaroa G, Rawlinson D, Featherstone L, Pitt M, Caly L, Druce J, et al. Direct RNA sequencing and early evolution of SARS-CoV-2. *bioRxiv* 2020:976167; doi:10.1101/2020.03.05.976167

18. Tombácz D, Balázs Z, Csabai Z, Moldován N, Szűcs A, Sharon D, et al. Characterization of the Dynamic Transcriptome of a Herpesvirus with Long-read Single Molecule Real-Time Sequencing. *Sci. Rep.* 2017;7:43751. doi:10.1038/srep43751

19. Miladi M, Fuchs J, Maier W, Weigang S, i Pedrosa ND, Weiss L, et al. The landscape of SARS-CoV-2 RNA modifications. *bioRxiv* 2020:204362; doi:10.1101/2020.07.18.204362

20. Maróti Z, Tombácz D, Prazsák I, Moldován N, Csabai Z, Torma G, et al. Time-course Transcriptome Analysis of Host Cell Response to Poxvirus Infection Using a Dual Long-read Sequencing Approach. *BMC Res. Notes* 2021;14:239. doi:10.1186/s13104-021-05657-x

21. Maróti Z, Tombácz D, Moldován N, Torma G, Jefferson VA, Csabai Z, et al. Long-read Time-course Profiling of the Host Cell Response to Herpesvirus Infection Using Nanopore and Synthetic Long-Read Transcriptome Sequencing. *Sci. Rep*. 2021;11:14219. doi: 10.1038/s41598-021-93142-7

22. Liao H, Cai D, Sun Y. VirStrain: a strain identification tool for RNA viruses. *Genome Biol*. 2022;23:38. doi:10.1186/s13059-022-02609-x

23. Li H. Minimap2: pairwise alignment for nucleotide sequences. *Bioinformatics* 2018;34:3094–3100. doi:10.1093/bioinformatics/bty191

24. Li H, Handsaker B, Wysoker A, Fennell T, Ruan J, Homer N, et al. The Sequence Alignment/Map format and SAMtools. *Bioinformatics* 2009;25:2078-9. doi:10.1093/bioinformatics/btp352

25. Moldován N. Seqtools: tools for NGS and third-generation sequencing (PacBio, Oxford Nanopore) data analysis. https://github.com/moldovannorbert/seqtools

26. Wickham H. ggplot2: Elegant Graphics for Data Analysis. 2nd ed. New York: Springer-Verlag; 2016.

27. Wickham et al., (2019). Welcome to the tidyverse. Journal of Open Source Software, 4(43), 1686, https://doi.org/10.21105/joss.01686

28. Morgan M, Pagès H, Obenchain V, Hayden N (2022). Rsamtools: Binary alignment (BAM), FASTA, variant call (BCF), and tabix file import. R package version 2.12.0, https://bioconductor.org/packages/Rsamtools

29. Lawrence M, Huber W, Pagès H, Aboyoun P, Carlson M, Gentleman R, Morgan M, Carey V (2013). "Software for Computing and Annotating Genomic Ranges." PLoS Computational Biology, 9. doi:10.1371/journal.pcbi.1003118, http://www.ploscompbiol.org/article/info%3Adoi%2F10.1371%2Fjournal.pcbi.1003118

30. Tidygenomics: Tidy Verbs for Dealing with Genomic Data Frames. https://const-ae.github.io/tidygenomics

31. Wickham H, François R, Henry L and Müller K (2022). dplyr: A Grammar of Data Manipulation. R package version 1.0.9. https://CRAN.R-project.org/package=dplyr

32. gggenes: a ggplot2 extension for drawing gene arrow maps. https://github.com/wilkox/gggenes

33. Kakuk B. Github repository of scripts used in "High Temporal-Resolution Nanopore Sequencing Dataset of SARS-CoV-2 and Host Cell RNAs". 2022 https://github.com/Balays/SARS-CoV2_ONT_data

34. Shafin K, Pesout T, Lorig-Roach R, Haukness M, Olsen HE, Bosworth C, et al. Nanopore sequencing and the Shasta toolkit enable efficient de novo assembly of eleven human genomes. *Nat Biotechnol*. 2020;38:1044–1053. doi:10.1038/s41587-020-0503-6

35. Aksamentov I., Roemer C., Hodcroft E. B., Neher R. A. Nextclade: clade assignment, mutation calling and quality control for viral genomes. *J. Open Source Softw.*, 2021;6:3773. doi: 10.21105/joss.037731

36. Weirather JL, de Cesare M, Wang Y, Piazza P, Sebastiano V, Wang X-J, et al. Comprehensive comparison of Pacific Biosciences and Oxford Nanopore Technologies and their applications to transcriptome analysis. *F1000Res*. 2017;6:100. doi:10.12688/f1000research.10571.2

37. Garalde DR, Snell EA, Jachimowicz D, Sipos B, Lloyd JH, Bruce M, et al. Highly parallel direct RNA sequencing on an array of nanopores. *Nat. Methods*. 2018;15:201–6. doi:10.1038/nmeth.4577

38. De Coster W, D’Hert S, Schultz DT, Cruts M, Van Broeckhoven C. NanoPack: visualizing and processing long-read sequencing data. *Bioinformatics* 2018;34:2666–2669. doi:10.1093/bioinformatics/bty149

39. Tardaguila M, de la Fuente L, Marti C, Pereira C, Pardo-Palacios FJ, del Risco H, et al. SQANTI: extensive characterization of long-read transcript sequences for quality control in full-length transcriptome identification and quantification. *Genome Res*. 2018;28:396-411. doi:10.1101/gr.222976.117

40. Ren J, Chaisson MJP. lra: A long read aligner for sequences and contigs. *PLoS Comput. Biol*. 2021;17:e1009078. doi:10.1371/journal.pcbi.1009078

41. Balázs Z, Tombácz D, Csabai Z, Moldován N, Snyder, Boldogkői Z. Template-switching artifacts resemble alternative polyadenylation. *BMC Genomics* 2019;20:824. doi:10.1186/s12864-019-6199-7

42. Balázs Z. LoRTIA: Long-read RNA-Seq Transcript Isoform Annotator toolkit. https://github.com/zsolt-balazs/LoRTIA

43. Amarasinghe SL, Ritchie ME, Gouil Q. long-read-tools.org: an interactive catalogue of analysis methods for long-read sequencing data. *GigaSci*. 2021;10:giab003. doi:10.1093/gigascience/giab003

44. Amarasinghe SL, Su S, Dong X, Zappia L, Ritchie ME, Gouil Q. Opportunities and challenges in long-read sequencing data analysis. *Genome Biol*. 2020;21:30. doi:10.1186/s13059-020-1935-5

45. Tombácz D, Dörmő A, Gulyás G, Csabai Z, Prazsák I, Kakuk B et al. Supporting data for "High Temporal-Resolution Nanopore Sequencing Dataset of SARS-CoV-2 and Host Cell RNAs" GigaScience Database. 2022 http://doi.org/10.5524/102256

Legend to Figures

Figure 1. Schematic representation of the workflow applied in this project. **A.** Isolation and detection of a Hungarian isolate of SARS-CoV-2 virus. The sample was collected from human nasopharyngeal swab. The SARS-CoV-2 infection was validated by RT-PCR using the RNA extracted from the sample. The virus was isolated from the sample and was maintained on Vero cells. **B.** Experimental workflow of the study. Vero cells were infected with SARS-CoV-2 and the cells were incubated at 37°C for 1, 2, 4, 6, 8, 10, 12, 14, 16, 18, 20, 24, 36, 48, 72 and 96 hours post-infection. Uninfected control cells were also propagated. Each time point experiment was carried out in three biological replicates. RNAs were purified from the samples, which was followed by the preparation of libraries and then sequencing using direct cDNA and direct RNA methods. Altogether, nine MinION flow cells (ONT) were used for this study. **C.** Bioinformatics workflow. The ONT's Guppy basecaller was used to identify the base sequence of the obtained reads, then they were aligned to the viral and host reference genomes by using the minimap2 mapper. Statistical data were generated with seqtools [25] and a custom R-workflow [33]. **D.** Quality of RNA samples were detected with a TapeStation 2200 system with RNA ScreenTape. TapeStation gel image shows that intact, high quality RNAs were isolated from the

samples and used for sequencing. The image shows the following samples: A1: marker; B1: 8h pi sample C; 12h pi sample A; 16h pi sample A; 18h pi sample B, 20h pi sample C; 36h pi sample A; 48h pi sample A; 96h pi sample B

Figure 2. Ratio of the sgRNAs to the gRNAs across the viral infection cycle in the dcDNA samples. The fitted loess function with 95% confidence intervals is shown in blue and gray, respectively.

Figure 3. Phylogenetic tree displays the sequenced SARS-CoV-2 strains, according to the designated clades of the virus. Our isolate is colored as red and a red arrow shows the position of our own isolate documented in the current study (OM812693.1). The position of the genome which was used as reference for aligning the reads (MT560672.1) is also indicated by a red arrow. The tree was generated by the Nextstrain pipeline. All variants are colored by their assigned clade, according to the nomenclature.

Figure 4. Whole genome coverage plot using high-quality (Q-score ≥8) reads from dcDNA samples which aligned to the SARS-CoV-2 genome used as references for this study. The coverages of the replicates from each hpi group were summed and the y-axes show the log2 of these values. Annotated protein-coding genes are shown at the bottom track. Direction of arrows depicts the coding strand.

Figure 5. Scatterplot of mean read lengths of the sequencing data derived from infected and uninfected samples, with 25% and 75% percentiles and a fitted loess function. **A.** Length of reads aligned to the viral **B.** and to the host genome. **C.** Read-length distribution of mock-infected samples mapped to the host genome.

Tables

Table 1. Summary data of the obtained read counts from dcDNA and dRNA sequencings. Low quality (failed) reads (Q-score <8) were filtered out from the passed reads (Q-score ≥8) by the MinKNOWs (Guppy, ONT) software.

Table 2. List of the sequences of barcodes used for multiplex sequencing. This table also contains the information about the barcoded samples loaded on the same flow cell. A, B, and C represent the biological replicates.

Additional Files

Supplementary Table S1. Summary statistics of the obtained reads from the infected samples. **A.** High quality reads, (Q-score ≥ 8). **B**. Low quality reads, (Q-score < 8).

Supplementary Table S2. Summary statistics of the obtained reads from the mock-infected samples. **A.** High quality reads, (Q-score ≥ 8). **B**. Low quality reads, (Q-score < 8).

Supplementary Table S3. Detailed information about the concentration of RNA and cDNA samples used for library preparation and sequencing. **A**. Concentration of RNA samples. Concentration of SARS-CoV-2 infected and mock-infected RNAs were measured with Qubit 4.0. The concentrations are in ng/µl. A, B and C represents the three biological replicates. **B**. Summary table of the poly(A)+ RNA concentrations. Concentrations of polyadenylated RNAs: from SARS-CoV-2 infected cells and from mock-infected cells in ng/µl. A, B and C represents the three biological replicates. **C**. The volume of polyA(+) RNA samples (100 ng) used for cDNA generation. A, B and C represents the three biological replicates. **D**. The amount (µl) of RNAs used for preparing a mixture for dRNA sequencing. Agencourt Ampure XP bead was used to get a higher concentration for the mixture (500 ng RNA in 9µl).

Supplementary Figure S1. Violin plot of mapped region length for genomic, sub-genomic and unclassified viral RNAs.

Supplementary Figure S2. VirStrain result showing the most probable strain present in the reads.

Supplementary Figure S3. Illustration of the ratio between SARS-CoV-2 and host cell read counts throughout the experiment. The viral read count was divided by the host read count for each replicate (group). The means and the standard deviations were also calculated and are shown with a straight line.

Supplementary Figure S4. Polar plot representation of sequencing coverages at the examined time points after viral infection (log10 scale).

Supplementary Figure S5. Line graph showing the virus and host read counts throughout the experiment.

Click here to access/download;Figure;Figure1.pdf

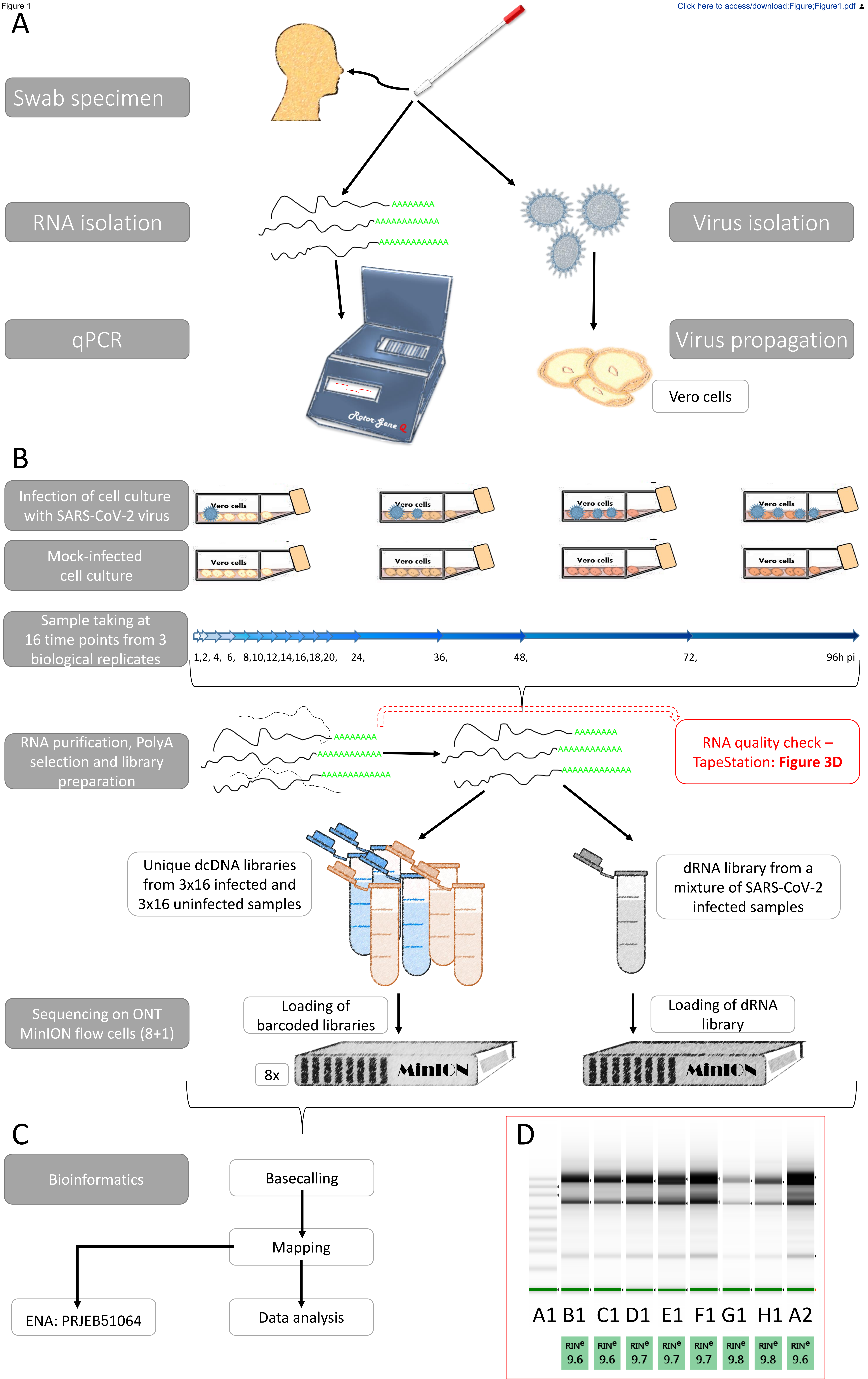

Figure 2

Click here to access/download;Figure;Figure2.pdf

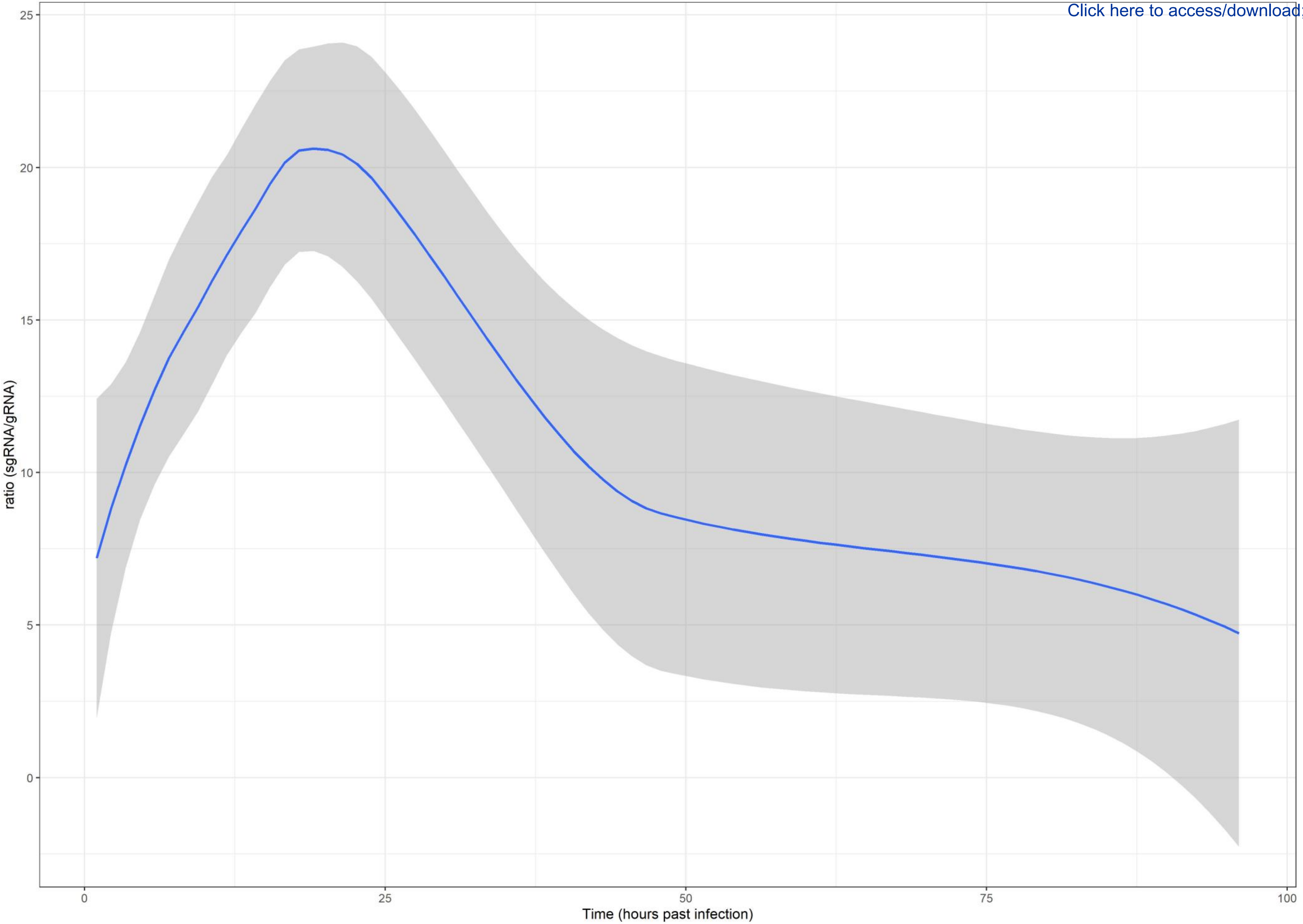

Figure 5

Click here to access/download;Figure;Figure5.pdf

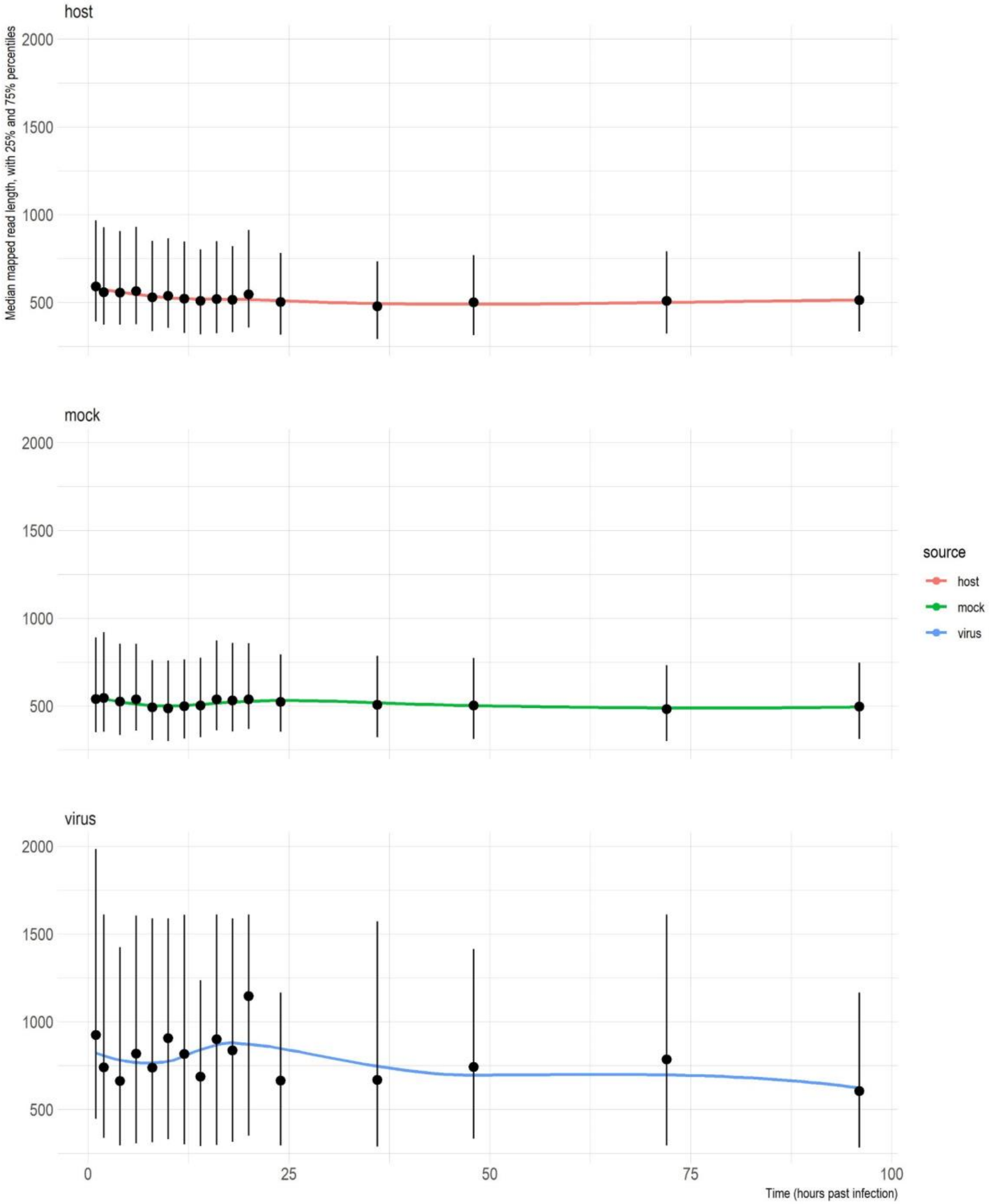

Supplementary Figure S1

Click here to access/download
Supplementary Material
SupplementaryFigure_S1.pdf

Supplementary Figure S2

Click here to access/download
Supplementary Material
SupplementaryFigure_S2.pdf

Supplementary Figure S3

Click here to access/download
Supplementary Material
SupplementaryFigure_S3.pdf

Supplementary Figure S4

Click here to access/download
Supplementary Material
SupplementaryFigure_S4.pdf

Supplementary Figure S5

Click here to access/download
Supplementary Material
SupplementaryFigure_S5.pdf

Supplementary Table S1

Click here to access/download

Supplementary Material

SupplementaryTableS1.xlsx

Supplementary Table S2

Click here to access/download
Supplementary Material
SupplementaryTableS2.xlsx

Supplementary Table S3

Click here to access/download
Supplementary Material
SupplementaryTableS3.xlsx
