# Supplementary figures and images for: High temporal resolution Nanopore sequencing dataset of SARS-CoV-2 and host cell RNAs
Source: Gigascience. 2022 Oct 17;11:giac094. doi: 10.1093/gigascience/giac094 (PMC9575581; doi:10.1093/gigascience/giac094)

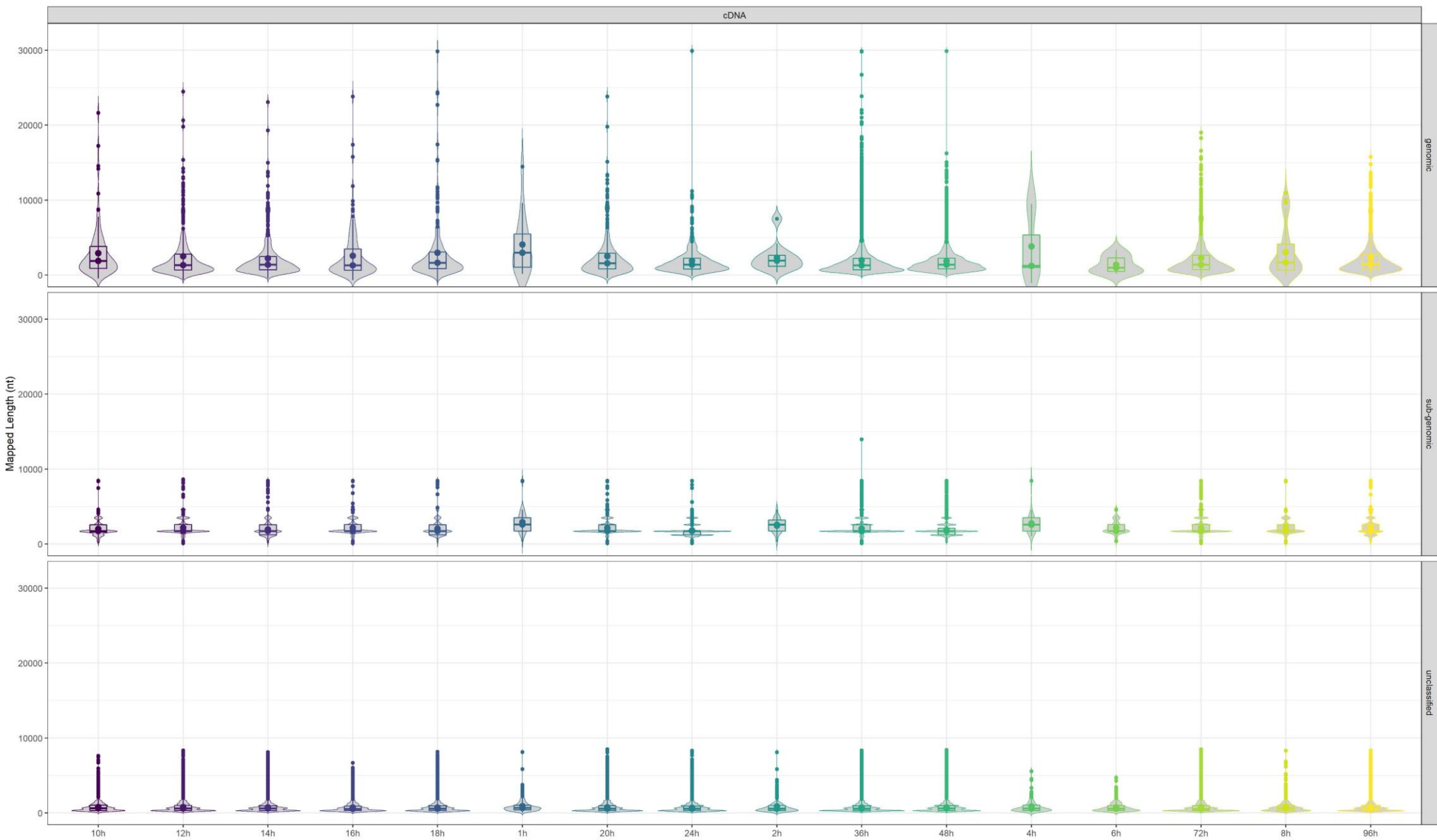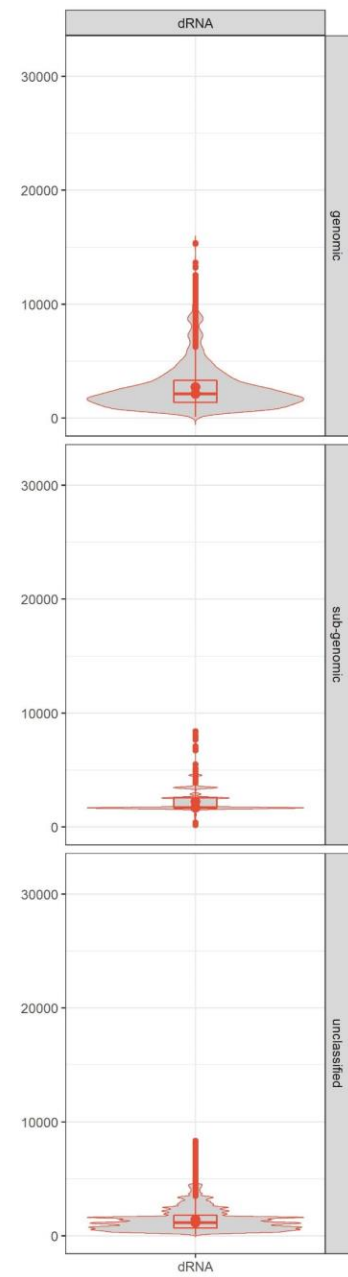

Supplement: giac094_Supplemental_Files [file giac094_supplemental_files.zip › SupplementaryFigure_S1.pdf]

Most Possible Strain: >MT560672.1

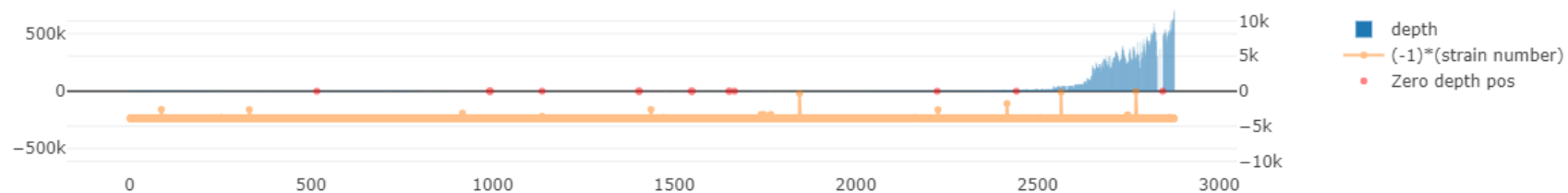

Other Possible Strain: >MT184913.1 (Rank:1)

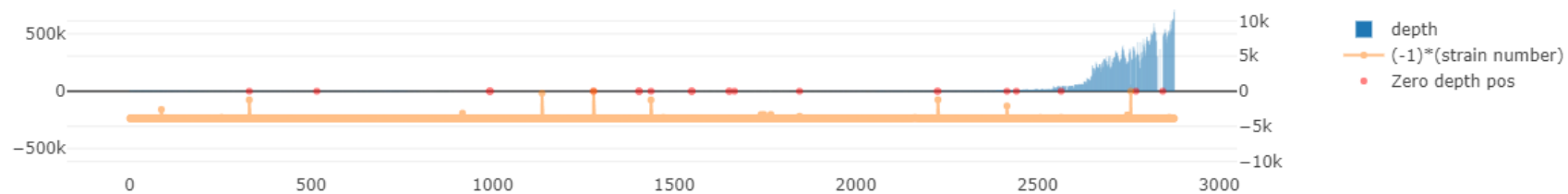

Other Possible Strain: >MT731667.1 (Rank:2)

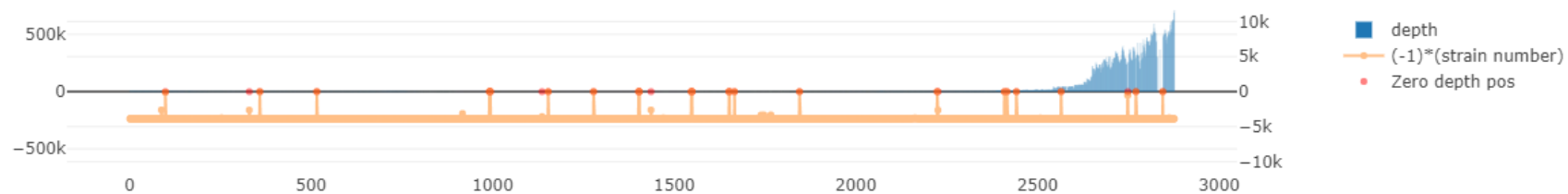

Supplement: giac094_Supplemental_Files [file giac094_supplemental_files.zip › SupplementaryFigure_S2.pdf]

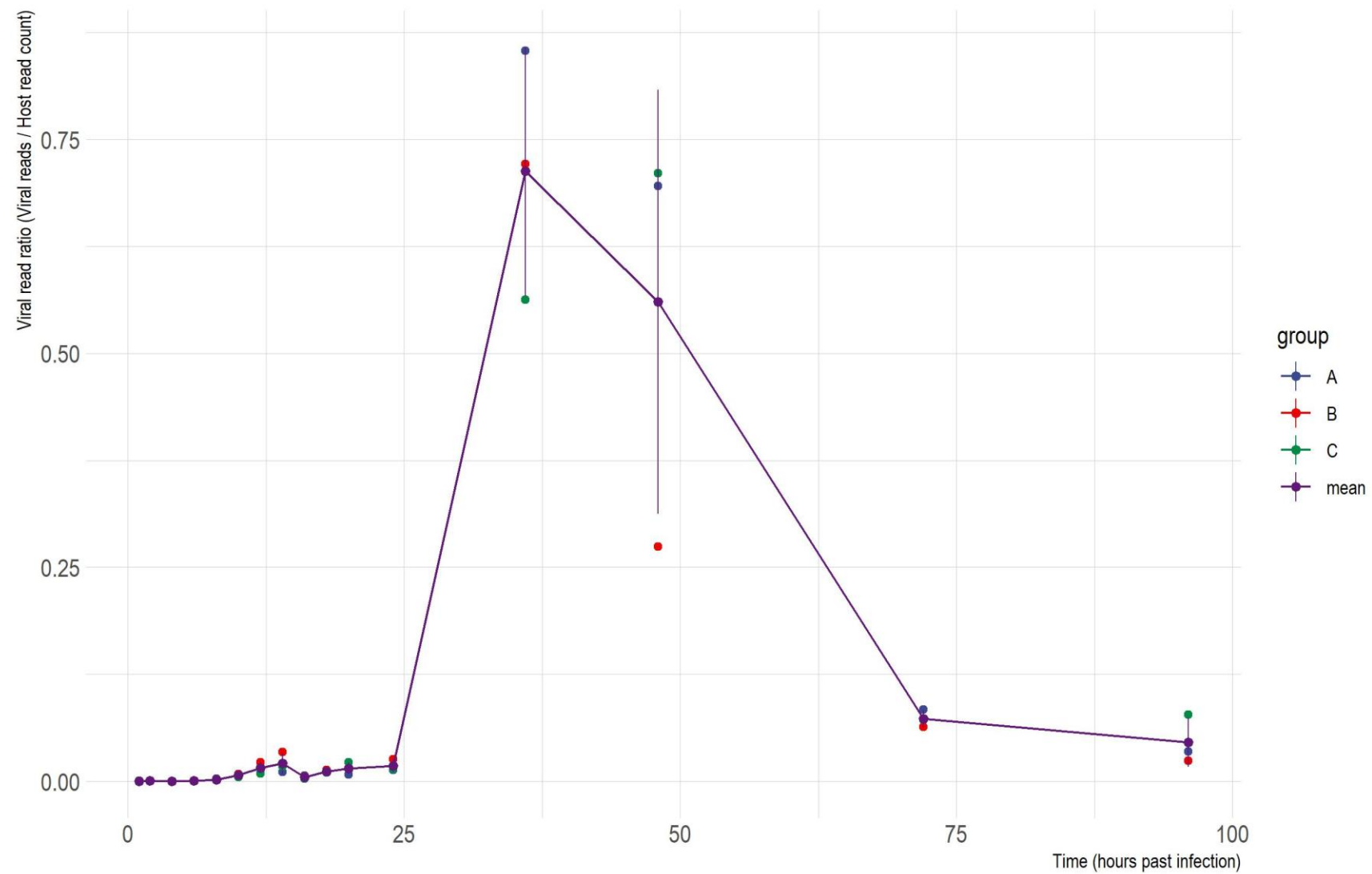

Supplement: giac094_Supplemental_Files [file giac094_supplemental_files.zip › SupplementaryFigure_S3.pdf]

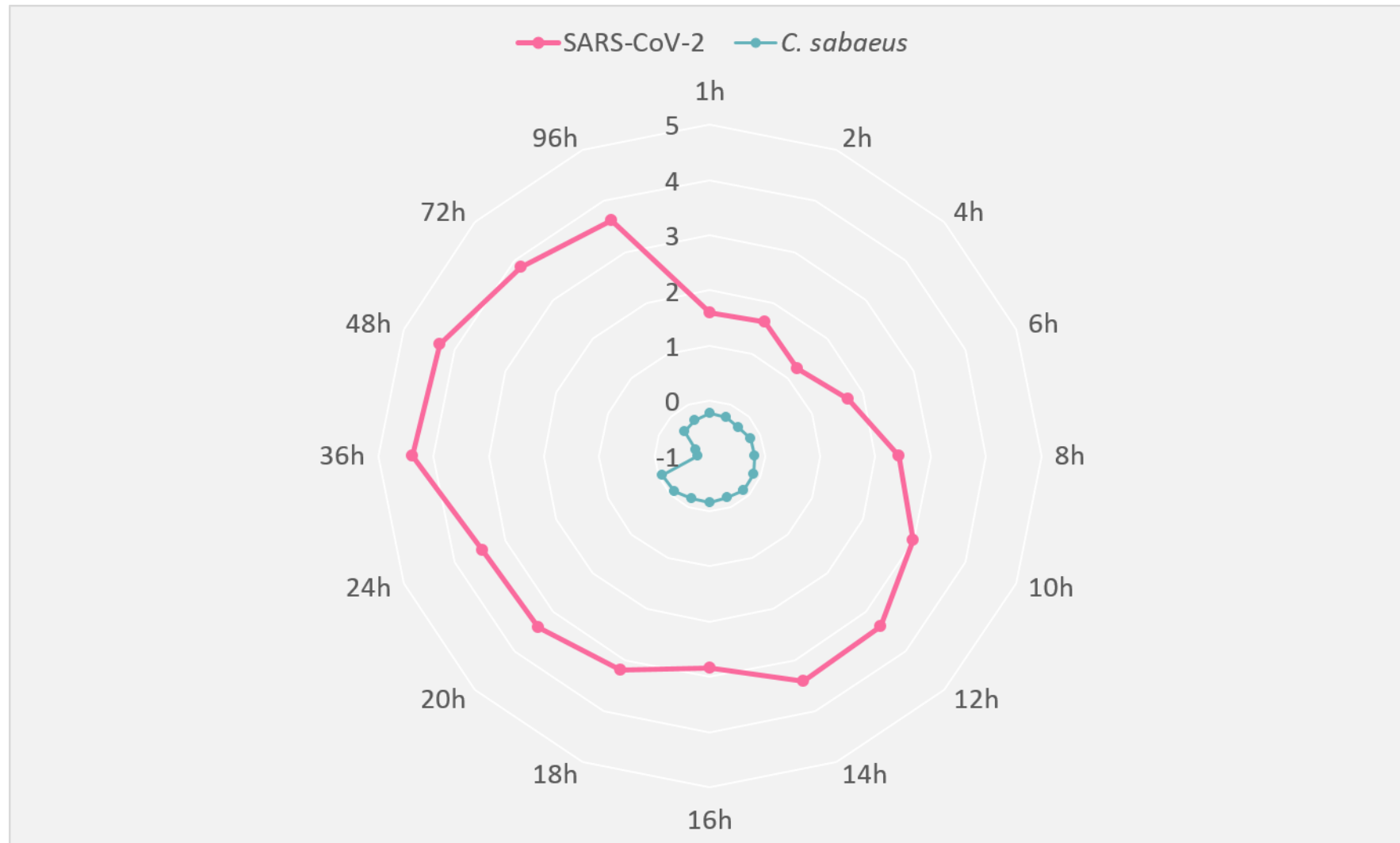

Supplement: giac094_Supplemental_Files [file giac094_supplemental_files.zip › SupplementaryFigure_S4.pdf]

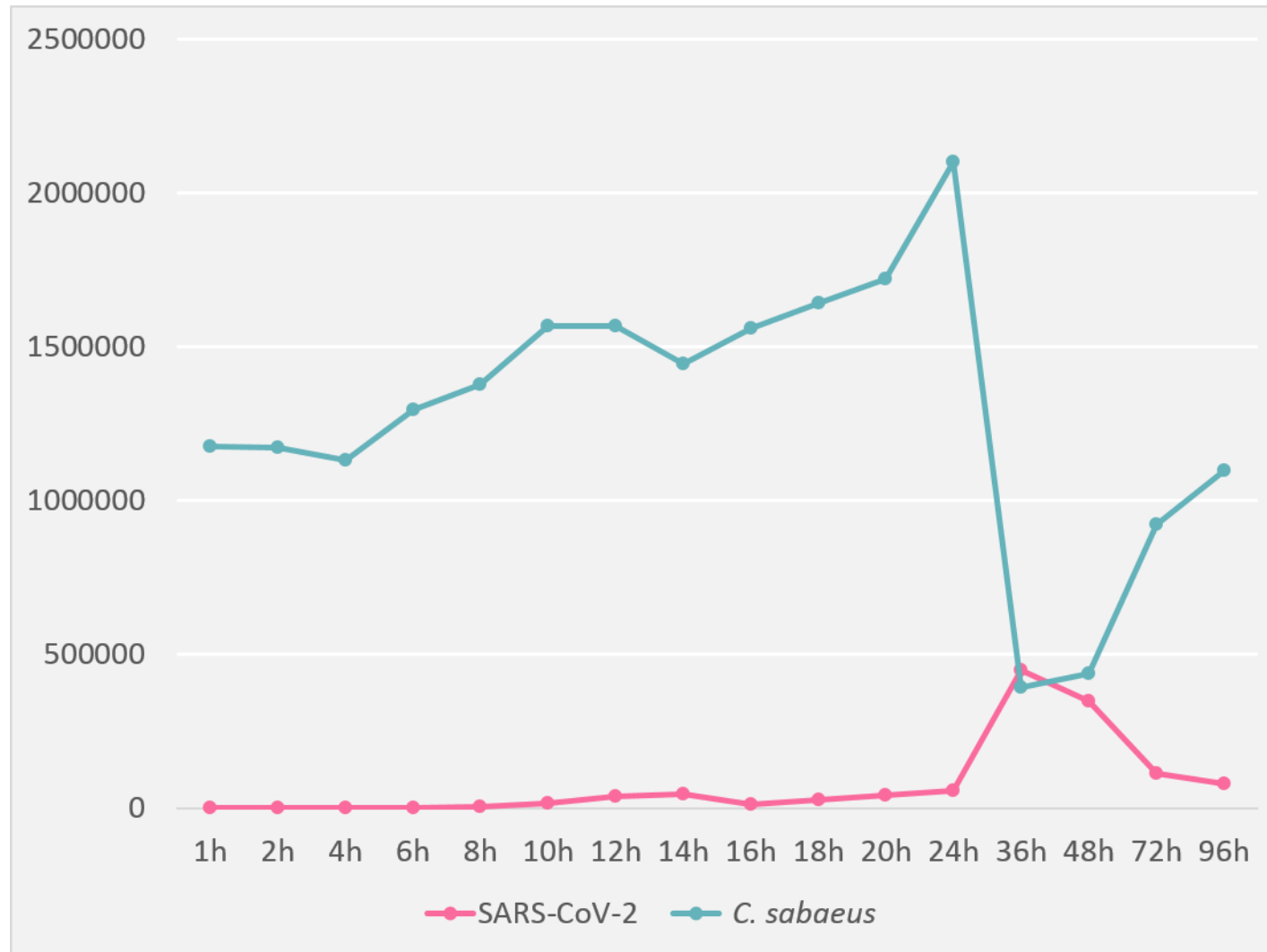

Supplement: giac094_Supplemental_Files [file giac094_supplemental_files.zip › SupplementaryFigure_S5.pdf]
