# Supplementary material for: High temporal resolution Nanopore sequencing dataset of SARS-CoV-2 and host cell RNAs
Source: Gigascience. 2022 Oct 17;11:giac094. doi: 10.1093/gigascience/giac094 (PMC9575581; doi:10.1093/gigascience/giac094)
Supplement: giac094_Response_to_Reviewer_Comments_Original_Submission [file giac094_response_to_reviewer_comments_original_submission.pdf]

Author's Response To Reviewer Comments

Close

Dear Editor,

We would like to thank the reviewers for their time spent on reviewing our manuscript and their helpful comments and suggestions. We have addressed and agreed with each comment below and hope it now satisfies the requirements for publication in GigaScience. Our point-by-point responses to the comments are listed below and the changes have been marked in blue in the revised version of the manuscript.

Reviewer #1:
In this work, Tombacz et al. provide a Nanopore RNA sequencing dataset of SARS-CoV-2 infected cells in several timepoints and sequencing setups. Both direct RNA-seq and cDNA-seq techniques have been utilized, and multiplex barcoded sequencing has been done for combining the samples. The dataset can be helpful to the community, such as for future transcriptomic studies of SARS-CoV-2, especially for studying the infection and expression dynamics. The text is well written and easy to follow. I find this work valuable; however, I can see several limitations in the analysis and representation of the results. Notably, the figures and tables representing statistical and biological insights of the data points are underworked, lack clarity, and provide limited information about the experiment. Further visualizations, analysis, and data processing could help to reveal the value and insights from this sequencing experiment.
We agree with all of the suggestions and revised the manuscript accordingly.

1. The presentation of reads coverage and lengths in Figs 1 & 2 are elementary, unpolished, and non-informative. Better annotation and labeling in Fig. 1 would be needed. Stacking so many violin plots in Fig 2 does not provide any valuable information and would only misguide. What are the messages of these figures? What do the authors expect the readers to catch from them? As noted, stacking many similar figures does not add further information. The authors may want to consider alternative representations and aggregation of the information, besides or replacing the current plots. For example, in Fig.2, scatter/line plots for the median & 25/75% percentile ranges, with an aggregation of the three replicates in on x-axis position, could help identify potential trends over the time points.

- Figures 1 and 2 (Figures 4 and 5 in the revised manuscript) have been changed, as suggested. In Figure 4, the three biological replicates of each time point have been merged. More details to the annotation have been added: the 16 non-structural proteins encoded by the ORF1a and 1b have been labelled. In Figure 5, The violin plots have been replaced by a line charts, and the 25/75% percentile ranges have also been indicated.

2. It is better to start the paper by presenting the current Fig.3 as the first one. This figure is the core of contributions and methodologies, and current Figs 1&2 are logical followups of this step.

- We have changed the order of the figures, as suggested.

3. There is a very limited description in the Figure Legends. The reader should be able to understand essential elements of the figures merely based on the Figure and its legend.

- Additional information have been added to the Figure Legends to improve the understanding.

4. This study does not provide much notable biological insight without demultiplexing the reads of each experimental condition into genomic and subgenomic subsets. Distinguishing the genomic and subgenomic reads and analyzing their relative ratio is essential in this temporal study. Due to the transcription process of coronaviruses, the genomic and subgenomic reads have very different characteristics, such as length distribution and cellular presence. Genomic and sub-genomic reads can be reliably identified by their coverage and splicing profiles, for enough long reads. It is essential that the authors further process the data by categorizing the genomic/subgenomic reads and the provide statistics such as read length for each category. It would also be interesting to observe the ratio of

genomic vs. subgenomic reads. This is an indicative metric of the infection state of the sample. An active infection has a higher sub-genomic share, while, e.g., a very early infection stage is expected to have a larger portion of genomic reads.

- The genomic and subgenomic reads have been identified and the temporal changes of their ratios were calculated and visualized (see details in the manuscripts). Due to the preference of the oligo(dT) primer-based long-read sequencing towards the short reads, the long reads are significantly underrepresented compared to the short ones. However, the changes in the ratios of subgenomic/genomic reads in time can provide important information on the replication and transcription of the virus.

5. Page-3: "[..] the nested set of subgenomic RNAs (sgRNAs) mapping to the 3'-third of the viral genome". Is 3'-third a typo? Otherwise, the text is not understandable.

- We have corrected the text.

6. Page-4: " because after a couple of hours, the virus can initiate a new infection cycle within the non-infected cells." More context and elaboration by citing some references can help to support the authors' claim. A gradual infection of non-infected cells can be assumed. However, "a couple of hours" and "initiate a new infection cycle" need further support in a scientific manuscript. The infection process is fairly gradual, but the wording here infers a sudden transition to infecting other cells only at a particular time point.

- We have modified the text and added a novel reference. We agree, the new infection is indeed gradual because the viral particles are continuously released from the infected cells. This phenomenon is discussed in the case of other viruses, such as in influenza virus:
https://www.ncbi.nlm.nih.gov/pmc/articles/PMC3840122/

7. Page-4: "[..]undergo alterations non-infected cells during the propagation therefore, we cannot decide whether the transcriptional changes in infected are due to the effect of the virus or to the time factor of culturing." This can be strong support for why this experiment has been done and for the value of this dataset. I would suggest mentioning this in the abstract to highlight the motivation.

- We have included this information to the abstract.

8. Page-4: "based studies have revealed a hidden transcriptional complexity in viruses [13,14]"
Besides Kim et. al, the first DRS experiments of coronaviruses have not been cited (doi.org/10.1101/gr.247064.118, doi.org/10.1101/2020.07.18.204362, doi.org/10.1101/2020.03.05.976167)

- The suggested references have been added to the revised manuscript.

9. Table-1: dcDNA is quite an uncommon term. In general, here and elsewhere in the text, insisting on a *direct* cDNA is a bit misleading. A "direct" cDNA sequencing is still an indirect sequencing of RNA molecules!

- We applied the terminology used by Oxford Nanopore Technologies in those sequencing when no PCR amplifications were applied. The terminology “direct (d)cDNA sequencing” is introduced by the Oxford Nanopore Technologies and it is commonly used for the non-amplified cDNA technique. See for example here: https://www.nature.com/articles/s41587-021-01108-x. Although, we agree with the reviewer that this terminology can be misleading since it is indeed not ‘direct’. We have added a definition to the text to clarify that the ‘direct cDNA sequencing’ is also termed as ‘non-amplified cDNA sequencing’.

10. Figs S2 and S3: Please also report the ratio of virus to host reads.

- A novel figure (Supplementary Figure S3) containing the requested data has been added to the revised manuscript. In the revised manuscript, Supplementary Figures S2 and S3 became Supplementary Figures S4 and S5, respectively.

Reviewer #2:
1. The authors provide a potentially useful dataset relating to transcripts from cultured SARS-CoV-2 material in a commonly used cell line (Vero). Relevant sequence data are publicly available and descriptions on the preparation of these data are for the most part detailed and adequate, although this

is lacking at times. Although the authors state that this dataset overcomes the limitations of available transcriptomic datasets, I do not believe this to be an accurate statement; based on comparable published work in this cell line, transcriptional activity is expected to peak at approximately one day post infection (Chang et al. 2021, Transcriptional and epi-transcriptional dynamics of SARS-CoV-2 during cellular infection), with the 96 hour period of infection described likely representing overlapping cellular infections of different stages. Secondly, many in the field have moved to use more appropriate cell lines in place of the Vero African Monkey kidney cell line, to better reflect changes in transcription during the course of infection in human and/or lung epithelial cells (See Finkel et al. 2020, The coding capacity of SARS-CoV-2). Lastly, the study would ideally be performed with a publicly available SARS-CoV-2 strain, as has been the case for earlier studies of this nature to allow for reproducibility and extension of the work presented by others. That said, the data are publicly available and could be of use.

- First of all, Chang and co-workers examined only three different time-points after infection (2, 24, and 48 hours post-infection). In contrast, we carried out a high temporal resolution experiment using 16 time points, which provides more precise information on the replication and transcription kinetics of SARS-CoV-2. Additionally, these authors used a low multiplicity of infection (MOI=0.1) for the infection, which allows the initiation of additional replication cycles at the late time points (24 and 48 hours post-infection). In our study we applied high MOI (5 pfu/cell) in order to avoid this possibility. Indeed, multiple cell lines are used for the studies of SARS-CoV-2 replication and transcription. Vero is a frequent choice, and therefore an appropriate model with respect of the reproducibility. Additionally, using various cell lines for the propagation of the virus can be useful for the better understanding the complexity and dynamics of SARS-CoV-2 transcriptome. We do not believe that, except some differences in the sequences, the transcriptomes of the various SARS-CoV-2 variants would differ significantly. However, if that were the case, it would be beneficial for better understanding the viral strategies.

Primary comments
2. I think that a statement detailing the ethics approval for this work would be essential, given materials used were collected from posthumously from a patient. Similarly, were these studies performed under appropriate containment, given classifications of SARS-CoV-2 at the time of the study?

- We have added the requested information to the revised manuscript.

3. I do not know what the authors mean in reference to a 'mixed time point sample' for the one direct RNA sample in this study; could this please be clarified?

- The text has been clarified.

Secondary comments
4. I believe the authors may over-simplify discontinuous extension of minus strands in saying that 'The gRNA and the sgRNAs have common 3'-termini since the RdRP synthesizes the positive sense RNAs from this end of the genome'. Each of the 5' and 3' sequence of gRNAs/sgRNAs are shared through this process of replication.

- We agree with this comment and the text has been corrected as suggested.

5. 'Infections are typically carried out using fresh, rapidly growing cells, and fresh cultures are also used as mock-infected cells. However, gene expression profiles may undergo alterations non-infected cells during the propagation therefore, we cannot decide whether the transcriptional changes in infected are due to the effect of the virus or to the time factor of culturing. This phenomenon is practically never tested in the experiments.' I do not follow what these sentences are referring to.

- We have modified the text to be more understandable. We note that the other Reviewer considered this experimental design so important that he/she recommended to include this information in the abstract.

6. 'Altogether, we generated almost 64 million long-reads, from which more than 1.8 million reads mapped to the SARS-CoV-2 and almost 48 million to the host reference genome, respectively (Table 1). The obtained read count resulted in a very high coverage across the viral genome (Figure 1). Detailed data on the read counts, quality of reads including read lengths (Figure 2), insertions, deletions, as well as mismatches are summarized in Supplementary Tables.' Could this perhaps be more appropriately placed in the data analysis section, rather than background?

- This part of the background section has been moved to the analysis part of the manuscript.

Close
