# Supplementary material for: High temporal resolution Nanopore sequencing dataset of SARS-CoV-2 and host cell RNAs
Source: Gigascience. 2022 Oct 17;11:giac094. doi: 10.1093/gigascience/giac094 (PMC9575581; doi:10.1093/gigascience/giac094)
Supplement: giac094_Response_to_Reviewer_Comments_Revision_1 [file giac094_response_to_reviewer_comments_revision_1.pdf]

Author's Response To Reviewer Comments

Close

Dear Hans,
Thank you for your feedback and suggestions.
We completed all the necessary corrections in the manuscript, including:
- provided reference for the GigaDB dataset
- omitted the Source code availability section
- removed highlights and tracking
- corrected the BioProject ID
- corrected references for supporting data
- provided numbered citations for every software used

We hope now the manuscript is apt for publication.

Close
