# Supplementary material for: High temporal resolution Nanopore sequencing dataset of SARS-CoV-2 and host cell RNAs
Source: Gigascience. 2022 Oct 17;11:giac094. doi: 10.1093/gigascience/giac094 (PMC9575581; doi:10.1093/gigascience/giac094)
Supplement: giac094_Reviewer_1_Report_Original_Submission — Milad Miladi -- 3/30/2022 Reviewed [file giac094_reviewer_1_report_original_submission.pdf]

Reviewer Report

Title: High Temporal-Resolution Nanopore Sequencing Dataset of SARS-CoV-2 and Host Cell RNAs

Version: Original Submission **Date:** 3/30/2022

Reviewer name: Milad Miladi

Reviewer Comments to Author:

In this work, Tombacz et al. provide a Nanopore RNA sequencing dataset of SARS-CoV-2 infected cells in several timepoints and sequencing setups. Both direct RNA-seq and cDNA-seq techniques have been utilized, and multiplex barcoded sequencing has been done for combining the samples.
The dataset can be helpful to the community, such as for future transcriptomic studies of SARS-CoV-2, especially for studying the infection and expression dynamics. The text is well written and easy to follow. I find this work valuable; however, I can see several limitations in the analysis and representation of the results. Notably, the figures and tables representing statistical and biological insights of the data points are underworked, lack clarity, and provide limited information about the experiment. Further visualizations, analysis, and data processing could help to reveal the value and insights from this sequencing experiment.
comments
1. The presentation of reads coverage and lengths in Figs 1 & 2 are elementary, unpolished, and non-informative. Better annotation and labeling in Fig. 1 would be needed. Stacking so many violin plots in Fig 2 does not provide any valuable information and would only misguide. What are the messages of these figures? What do the authors expect the readers to catch from them?
As noted, stacking many similar figures does not add further information. The authors may want to consider alternative representations and aggregation of the information, besides or replacing the current plots. For example, in Fig.2, scatter/line plots for the median & 25/75% percentile ranges, with an aggregation of the three replicates in on x-axis position, could help identify potential trends over the time points.
2. It is better to start the paper by presenting the current Fig.3 as the first one. This figure is the core of contributions and methodologies, and current Figs 1&2 are logical followups of this step.
3. There is a very limited description in the Figure Legends. The reader should be able to understand essential elements of the figures merely based on the Figure and its legend.
4. This study does not provide much notable biological insight without demultiplexing the reads of each experimental condition into genomic and subgenomic subsets.
Distinguishing the genomic and subgenomic reads and analyzing their relative ratio is essential in this temporal study. Due to the transcription process of coronaviruses, the genomic and subgenomic reads have very different characteristics, such as length distribution and cellular presence. Genomic and sub-genomic reads can be reliably identified by their coverage and splicing profiles, for enough long reads.
It is essential that the authors further process the data by categorizing the genomic/subgenomic reads and the provide statistics such as read length for each category. It would also be interesting to observe the ratio of genomic vs. subgenomic reads. This is an indicative metric of the infection state of the

sample. An active infection has a higher sub-genomic share, while, e.g., a very early infection stage is expected to have a larger portion of genomic reads.
5. Page-3: "[..] the nested set of subgenomic RNAs (sgRNAs) mapping to the 3'-third of the viral genome". Is 3'-third a typo? Otherwise, the text is not understandable.
6. Page-4: " because after a couple of hours, the virus can initiate a new infection cycle within the non-infected cells."
More context and elaboration by citing some references can help to support the authors' claim. A gradual infection of non-infected cells can be assumed. However, "a couple of hours" and "initiate a new infection cycle" need further support in a scientific manuscript. The infection process is fairly gradual, but the wording here infers a sudden transition to infecting other cells only at a particular time point.
7. Page-4: "[..]undergo alterations non-infected cells during the propagation therefore, we cannot decide whether the transcriptional changes in infected are due to the effect of the virus or to the time factor of culturing."
This can be strong support for why this experiment has been done and for the value of this dataset. I would suggest mentioning this in the abstract to highlight the motivation.
8. Page-4: "based studies have revealed a hidden transcriptional complexity in viruses [13,14]"
Besides Kim et. al, the first DRS experiments of coronaviruses have not been cited (doi.org/10.1101/gr.247064.118, doi.org/10.1101/2020.07.18.204362, doi.org/10.1101/2020.03.05.976167)
9. Table-1: dcDNA is quite an uncommon term. In general, here and elsewhere in the text, insisting on a *direct* cDNA is a bit misleading. A "direct" cDNA sequencing is still an indirect sequencing of RNA molecules!
10. Figs S2 and S3: Please also report the ratio of virus to host reads.

Level of Interest

Please indicate how interesting you found the manuscript: Choose an item.

Quality of Written English

Please indicate the quality of language in the manuscript: Choose an item.

Declaration of Competing Interests

Please complete a declaration of competing interests, considering the following questions:

- Have you in the past five years received reimbursements, fees, funding, or salary from an organisation that may in any way gain or lose financially from the publication of this manuscript, either now or in the future?
- Do you hold any stocks or shares in an organisation that may in any way gain or lose financially from the publication of this manuscript, either now or in the future?

- Do you hold or are you currently applying for any patents relating to the content of the manuscript?
- Have you received reimbursements, fees, funding, or salary from an organization that holds or has applied for patents relating to the content of the manuscript?
- Do you have any other financial competing interests?
- Do you have any non-financial competing interests in relation to this paper?

If you can answer no to all of the above, write 'I declare that I have no competing interests' below. If your reply is yes to any, please give details below.

I declare that I have no competing interests

I agree to the open peer review policy of the journal. I understand that my name will be included on my report to the authors and, if the manuscript is accepted for publication, my named report including any attachments I upload will be posted on the website along with the authors' responses. I agree for my report to be made available under an Open Access Creative Commons CC-BY license (http://creativecommons.org/licenses/by/4.0/). I understand that any comments which I do not wish to be included in my named report can be included as confidential comments to the editors, which will not be published.

Choose an item.

To further support our reviewers, we have joined with Publons, where you can gain additional credit to further highlight your hard work (see: https://publons.com/journal/530/gigascience). On publication of this paper, your review will be automatically added to Publons, you can then choose whether or not to claim your Publons credit. I understand this statement.

Yes Choose an item.
