# Supplementary material for: High temporal resolution Nanopore sequencing dataset of SARS-CoV-2 and host cell RNAs
Source: Gigascience. 2022 Oct 17;11:giac094. doi: 10.1093/gigascience/giac094 (PMC9575581; doi:10.1093/gigascience/giac094)
Supplement: giac094_Reviewer_2_Report_Original_Submission — George Taiaroa -- 4/15/2022 Reviewed [file giac094_reviewer_2_report_original_submission.pdf]

Reviewer Report

Title: High Temporal-Resolution Nanopore Sequencing Dataset of SARS-CoV-2 and Host Cell RNAs

Version: Original Submission **Date:** 4/15/2022

Reviewer name: George Taiaroa

Reviewer Comments to Author:

The authors provide a potentially useful dataset relating to transcripts from cultured SARS-CoV-2 material in a commonly used cell line (Vero). Relevant sequence data are publicly available and descriptions on the preparation of these data are for the most part detailed and adequate, although this is lacking at times. Although the authors state that this dataset overcomes the limitations of available transcriptomic datasets, I do not believe this to be an accurate statement; based on comparable published work in this cell line, transcriptional activity is expected to peak at approximately one day post infection (Chang et al. 2021, Transcriptional and epi-transcriptional dynamics of SARS-CoV-2 during cellular infection), with the 96 hour period of infection described likely representing overlapping cellular infections of different stages. Secondly, many in the field have moved to use more appropriate cell lines in place of the Vero African Monkey kidney cell line, to better reflect changes in transcription during the course of infection in human and/or lung epithelial cells (See Finkel et al. 2020, The coding capacity of SARS-CoV-2). Lastly, the study would ideally be performed with a publicly available SARS-CoV-2 strain, as has been the case for earlier studies of this nature to allow for reproducibility and extension of the work presented by others. That said, the data are publicly available and could be of use.
Primary comments
I think that a statement detailing the ethics approval for this work would be essential, given materials used were collected from posthumously from a patient. Similarly, were these studies performed under appropriate containment, given classifications of SARS-CoV-2 at the time of the study?
I do not know what the authors mean in reference to a 'mixed time point sample' for the one direct RNA sample in this study; could this please be clarified?
Secondary comments
I believe the authors may over-simplify discontinuous extension of minus strands in saying that 'The gRNA and the sgRNAs have common 3'-termini since the RdRP synthesizes the positive sense RNAs from this end of the genome'. Each of the 5' and 3' sequence of gRNAs/sgRNAs are shared through this process of replication.
'Infections are typically carried out using fresh, rapidly growing cells, and fresh cultures are also used as mock-infected cells. However, gene expression profiles may undergo alterations non-infected cells during the propagation therefore, we cannot decide whether the transcriptional changes in infected are due to the effect of the virus or to the time factor of culturing. This phenomenon is practically never tested in the experiments.' I do not follow what these sentences are referring to.
'Altogether, we generated almost 64 million long-reads, from which more than 1.8 million reads mapped to the SARS-CoV-2 and almost 48 million to the host reference genome, respectively (Table 1). The obtained read count resulted in a very high coverage across the viral genome (Figure 1). Detailed data

on the read counts, quality of reads including read lengths (Figure 2), insertions, deletions, as well as mismatches are summarized in Supplementary Tables.' Could this perhaps be more appropriately placed in the data analysis section, rather than background?

Level of Interest

Please indicate how interesting you found the manuscript: Choose an item.

Quality of Written English

Please indicate the quality of language in the manuscript: Choose an item.

Declaration of Competing Interests

Please complete a declaration of competing interests, considering the following questions:

- Have you in the past five years received reimbursements, fees, funding, or salary from an organisation that may in any way gain or lose financially from the publication of this manuscript, either now or in the future?
- Do you hold any stocks or shares in an organisation that may in any way gain or lose financially from the publication of this manuscript, either now or in the future?
- Do you hold or are you currently applying for any patents relating to the content of the manuscript?
- Have you received reimbursements, fees, funding, or salary from an organization that holds or has applied for patents relating to the content of the manuscript?
- Do you have any other financial competing interests?
- Do you have any non-financial competing interests in relation to this paper?

If you can answer no to all of the above, write 'I declare that I have no competing interests' below. If your reply is yes to any, please give details below.

I declare that I have no competing interests

I agree to the open peer review policy of the journal. I understand that my name will be included on my report to the authors and, if the manuscript is accepted for publication, my named report including any attachments I upload will be posted on the website along with the authors' responses. I agree for my report to be made available under an Open Access Creative Commons CC-BY license (http://creativecommons.org/licenses/by/4.0/). I understand that any comments which I do not wish to be included in my named report can be included as confidential comments to the editors, which will not be published.

Choose an item.

To further support our reviewers, we have joined with Publons, where you can gain additional credit to further highlight your hard work (see: https://publons.com/journal/530/gigascience). On publication of this paper, your review will be automatically added to Publons, you can then choose whether or not to claim your Publons credit. I understand this statement.

Yes Choose an item.
